# Recent Progress in MXene-Based Materials for Supercapacitors and Electrochemical Sensing Applications

**DOI:** 10.3390/bios15050288

**Published:** 2025-05-03

**Authors:** Khursheed Ahmad, Tae Hwan Oh

**Affiliations:** School of Chemical Engineering, Yeungnam University, Gyeongsan 38541, Republic of Korea

**Keywords:** MXenes, biosensors, SCs, sensors, hybrid materials

## Abstract

In recent years, MXene-based materials have received extensive interest for a variety of applications, including energy storage, solar cells, sensors, photo-catalysis, etc., due to their extraordinary optoelectronic and physicochemical properties. MXene-based electrode materials exhibit excellent electrochemical properties for supercapacitors (SCs) and electrochemical sensing technologies due to the presence of acceptable electrocatalytic characteristics. Herein, we reviewed publications from recent years on the development of MXenes and their composites for SCs and electrochemical sensors. MXene-based materials with polymers, metal oxides, metal sulfides or selenides; metal–organic frameworks (MOFs); layered double hydroxides (LDHs); and carbon-based materials such as graphene, carbon nanotubes, etc., have been reviewed for their potential applications in SCs. MXene-based hybrid composites have also been reviewed for electrochemical sensing applications. Furthermore, challenges and future perspectives are discussed. It is expected that the present article will be beneficial for scientists working on the modification of MXene-based materials for SCs and electrochemical sensing technologies.

## 1. Introduction

In the previous few decades, layered two-dimensional (2D) materials dominated the field of materials science due to their unique and extraordinary electrochemical, electrical, mechanical and optoelectronic characteristics [1]. In 2011, Naguib et al. [2] discovered a new 2D layered material, e.g., metal carbide. This layered 2D family has been named as MXene [3,4,5]. MXenes are generally formed by the etching method. Typically, the titanium aluminum carbide (Ti_3_AlC_2_) MAX phase is converted to the Ti_3_C_2_ MXene phase via the etching method [6]. The reported literature shows that MXenes have excellent conducting properties and acceptable thermal stability with decent optoelectronic and electrochemical properties [7,8,9,10]. In previous years, MXene-based hybrid electrode materials have been widely used for various applications, including dye-sensitized solar cells [11], sensors [12], oxygen evolution reactions (OERs) [13], hydrogen evolution reactions (HERs) [14], batteries [15], perovskite solar cells [16] and catalysis [17].

The energy crisis is one of the major threats to the world and needs significant attention from the scientific community [18]. In the current scenario, it is found that present energy requirements depend on fossil fuels and conventional energy sources [19,20]. Unfortunately, fossil fuels are limited and may be responsible for environmental pollution [21]. Thus, it is of great importance to utilize and explore renewable energy sources to overcome the issue of the energy crisis. Previously, rapid growth in the publication of research articles on energy storage devices has been noticed, which can significantly contribute to alleviating the energy crisis [22]. Super-capacitors (SCs) are energy storage devices that may bridge the gap between batteries and conventional capacitors [23,24,25]. In recent years, metal sulfides [26], metal oxides [27], polymers [28], graphene [29], carbon nitride [30], MXenes [31] and layered double hydroxide (LDH) [32] materials have been widely used for SC applications. MXene-based materials exhibit excellent electrochemical performance and charge storage properties due to their layered structure and high conductivity [33]. MXene-based materials are also a promising candidate for electrochemical sensing technology [34,35,36]. Electrochemical sensors are the new generation of sensing technology, which can significantly monitor the presence of biomolecules and toxic substances using voltammetry and amperometric methods [37,38,39,40]. We have reviewed the progress in the fabrication of MXene-based materials for SCs and electrochemical sensing applications of various analytes. We believed that a brief summary of the use of MXene-based materials for SCs and electrochemical sensors may benefit researchers who are currently involved in the development of MXene-based SCs and electrochemical sensing devices.

This review article compiled the previously reported progress on MXene-based composites with metal oxides, metal sulfides/selenides, polymers, MOFs, LDHs, etc., for SCs and sensing applications.

## 2. Synthetic Procedures and Properties of MXene

### 2.1. Etching Approaches (Direct HF, In Situ HF and Molten Salt Etching)

In the previous years, etching was found to be the most efficient approach for the conversion of the MAX phase to MXene. HF (hydrofluoric) acid is a highly efficient and strong etching agent for the preparation of MXene. In a previous study, Naguib et al. [5] used 2000 mg of titanium aluminum carbide (Ti_3_AlC_2_) as the precursor and added it to 0.4 L of a solution of HF. It can be noted that HF has several negative impacts on human life due to its corrosive nature. It is also necessary to take serious precautions before handling it. Thus, a mild HF etching method was developed for the preparation of MXene. In this regard, an in situ HF etching method was developed and utilized for the preparation of MXene. Ghidiu et al. [3] adopted lithium fluoride (LiF) and hydrochloric acid (HCl) as etching agents. The authors dissolved LiF in the 6 M HCl solution, Ti_3_AlC_2_ was added to the above-mentioned solution and the reaction mixture was maintained at 40 °C. The obtained precipitate was filtered and repeatedly washed with DI water several times. Although this method has advantages, such as mild HF conditions or in situ formation of HF, it is relatively better compared to the direct use of concentrated HF. But the in situ etching approach is less efficient compared to the direct HF etching method. Recently, alkali etching [4] and molten salt etching [8] methods were also developed, but their performance needs to be improved for the uniform formation of layered MXene.

### 2.2. Hydrothermal/Solvothermal Approaches

Hydrothermal and solvothermal methods are efficient and promising synthetic procedures for the preparation of metal oxides. Previously, the hydrothermal method was adopted by Li et al. [6] for the preparation of MXene. In brief, 0.1 g of Ti_3_AlC_2_ was added to 25 mL of sodium hydroxide (NaOH) solution and subjected to hydrothermal conditions (65 °C for 12 h) for the preparation of MXene. After the hydrothermal reactor cooled down, the MXene sample was collected.

### 2.3. Other Methods

Previously, exfoliation and electrochemical etching methods also received significant attention because of their promising feature and cost-effectiveness. In the case of the exfoliation method, various intercalants, such as tetrabutylammonium hydroxide (TBAOH), are used for the preparation of MXene. The TBAOH-based study showed that few-layered MXenes are formed, whereas NH_4_HF_2_-based investigations suggested the formation of multilayered MXenes [35]. Li et al. [15] used an electrochemical method for the preparation of MXenes. Despite the several advantages of exfoliation and electrochemical methods, it is necessary to develop other novel green synthetic methods for the synthesis of MXenes.

## 3. SC Applications

### 3.1. MXenes and Functionalized/Doped MXenes Based SCs

**Figure 1:**
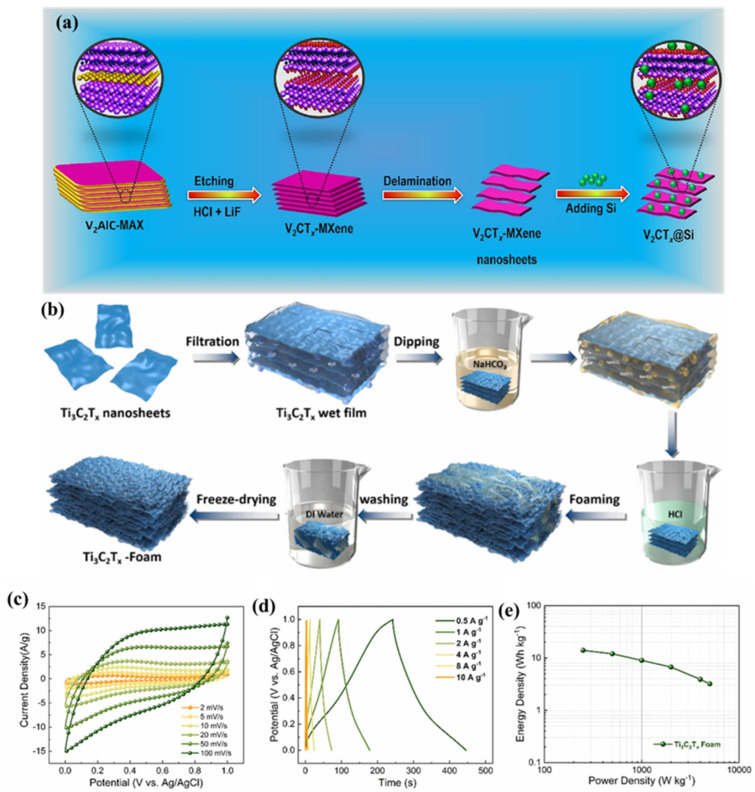
Schematic picture of the preparation of (**a**) V_2_CT_x_@Si [43] and (**b**) Ti_3_C_2_T*_x_* foam [45]. (**c**) CV of Ti_3_C_2_T*_x_* foam at varied scan rates. (**d**) GCD data of Ti_3_C_2_T*_x_* foam at different current densities [45]. (**e**) Ragone plots show relation between energy density and power density of Ti_3_C_2_T*_x_* foam-based SCs [45]. Reproduced with permission from references [43,45].

MXenes have excellent properties, such as electrical conductivity, magnetic properties, high surface area and decent electrochemical properties. These properties make them a desirable candidate for electrochemical applications, such as SCs and sensors. In previous studies, a large number of research articles have been published on the use of MXene-based materials for the construction of SCs. Yuan et al. [41] proposed the construction of additive-free MXene sediment ink for the development of SCs. The proposed SCs demonstrated a decent areal capacitance of 2.337 F/cm^2^ and retained excellent capacitance after 10,000 cycles. This work indicated that waste-free MXene ink with three-dimensional (3D) technology may be a great idea for the development of high-performance flexible SCs for large-scale applications. Wang et al. [42] explored the construction of MXene cross-linked organic gel electrolyte (OGE)-based SCs. The proposed SCs have acceptable stability with decent electrochemical performance. Mateen et al. [43] fabricated vanadium carbide MXene (V_2_CT*_x_*)- and silicon (Si)-based SCs and observed that V_2_CT*_x_*@Si improved electrochemical properties, storage capability and conductivity. The preparation of the proposed V_2_CT*_x_*@Si has been explained in the schematic picture in Figure 1a. It can be seen from the schematic illustration that V_2_CT*_x_*@Si involves an etching step using hydrochloride (HCl) and lithium fluoride (LiF) and a delamination process, which yields V_2_CT*_x_* MXene nanosheets. Furthermore, Si was incorporated and V_2_CT*_x_*@Si was formed. The obtained V_2_CT*_x_*@Si was characterized by various physicochemical methods, including photoelectron X-ray spectroscopy (XPS); field emission scanning electron microscopy (FESEM); X-ray diffraction (XRD); and transmission electron spectroscopy (TEM) methods. After the confirmation of the formation of V_2_CT*_x_* MXene nanosheets, the authors conducted electrochemical measurements for SC studies by utilizing electrochemical impedance spectroscopy (EIS); cyclic voltammetry (CV); and galvanostatic charge–discharge (GCD) methods. The electrochemical studies showed that V_2_CT*_x_*@Si has decent electrical conductivity and energy storage properties and can be explored as a potential candidate for SCs. Thus, the proposed V_2_CT*_x_*@Si-based SCs exhibit a specific capacitance (C_sp_) of 557.7 F/g at 1A/g and 10,000 stability cycles. Mu et al. [44] presented the rational design of a new, efficient and suitable nickel phthalocyanine (NiPc)-reinforced MXene electrode for SCs. The authors reported a C_sp_ of 792 F/g (1/g) with 5000 cycles for the proposed SCs. The improved C_sp_ of the proposed SCs may be ascribed to the introduction of NiPc into the MXene matrix, which may have efficiently reduced the restacking/agglomeration of the MXene sheets and increased the interlayer spacing and ion transfer rate. Liu et al. [45] reported a novel chemical foaming method to convert Ti_3_C_2_T_x_ wet films into 3D porous foams and employed it in SCs. Figure 1b shows the preparation of Ti_3_C_2_T*_x_* foam. After confirming the formation of Ti_3_C_2_T*_x_* foam, the authors employed it as an energy storage material for its application in SCs. It was observed that the prepared Ti_3_C_2_T_x_ foam exhibits a decent specific surface area of 60.8 m^2^/g with good porosity. The CV results of the Ti_3_C_2_T*_x_* foam at different scan rates are presented in Figure 1c. It was seen that the CV results show the acceptable electrochemical activity of the Ti_3_C_2_T*_x_* foam for SCs. Therefore, GCD curves of the Ti_3_C_2_T*_x_* foam were recorded, and the authors observed that Ti_3_C_2_T*_x_* foam shows a low Csp at 10 A/g and the highest Csp value at 0.5 A/g (Figure 1d). The authors achieved a Csp of 426 F/g (1 A/g) with 10,000 stability cycles. The energy density/power density relation for Ti_3_C_2_T*_x_* foam-based SCs is displayed in Figure 1e. It was assumed that the foaming method may have created a 3D porous structure, which improves ion accessibility and active sites, and improved electrochemical performance was observed.

**Figure 2:**
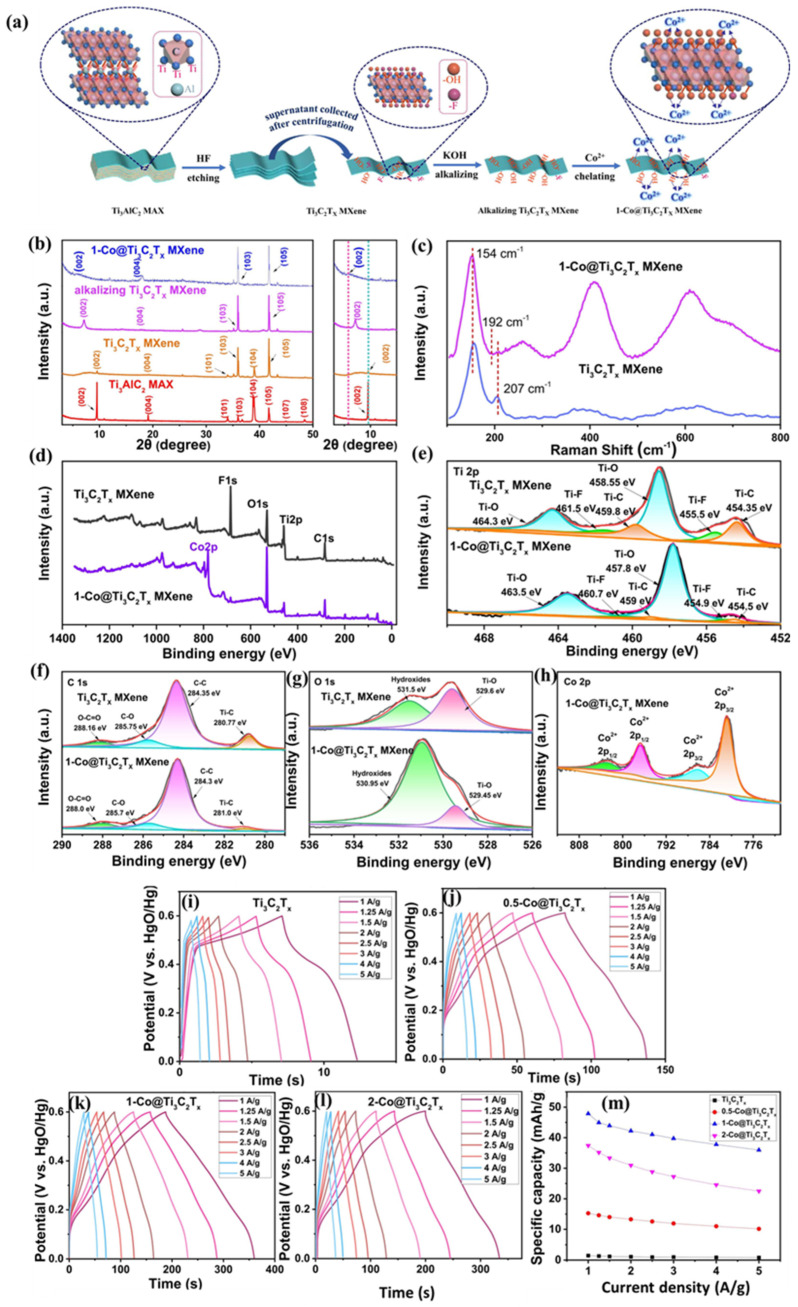
(**a**) Schematic graph shows the formation of Co@Ti_3_C_2_T_x_. (**b**) XRD of Ti_3_C_2_T_x_ MAX, Ti_3_C_2_T_x_ MXene, alkalizing Ti_3_C_2_T_x_ MXene and 1-Co@Ti_3_C_2_T_x_ MXene. (**c**) Raman spectra of Ti_3_C_2_T_x_ and 1-Co@Ti_3_C_2_T_x_. (**d**) XPS spectra of Ti_3_C_2_T_x_ and 1-Co@Ti_3_C_2_T_x_. High-resolution XPS of (**e**) Ti2p, (**f**) C1s, (**g**) O1s and (**h**) Co2p. GCD curves of (**i**) Ti_3_C_2_T_x_, (**j**) 0.5-Co@Ti_3_C_2_T_x_, (**k**) 1-Co@Ti_3_C_2_T_x_ and (**l**) 2-Co@Ti_3_C_2_T_x_ at varied current densities. (**m**) Specific capacity versus current density curves of different electrode materials. Reproduced with permission from reference [51].

In another report, Cai et al. [46] proposed the preparation of nitrogen (N)-doped Ti_3_C_2_T_x_ for its application in SCs. The N-Ti_3_C_2_T_x_-based SCs show a C_sp_ of 449 F/g at 2 mV/s with 8000 stability cycles, which is higher than pristine Ti_3_C_2_T_x_ (321 F/g)-based SCs. The authors found that N-doping increased layer spacing and reduced fluorine (F) in N-Ti_3_C_2_T_x_, which significantly improved ion diffusion and the accessibility of active sites and enhanced the C_sp_ and stability at N-Ti_3_C_2_T_x_-based SCs. This work presented new possibilities for researchers to develop doping strategies for the preparation of Ti_3_C_2_T_x_ for SCs. Yang et al. [47] adopted niobium-based MXene (Nb_2_C), formed its composite with o-doped porous biomass carbon and reported a Csp of 465.6 F/g in 6 molar (M) potassium hydroxide (KOH). In another study, Wang et al. [48] obtained a Csp of 1014 F/cm^3^ at 2 mV/s for Ti_2.9_Nb_0.1_C_2_T*_x_*-based SCs. The authors stated that Nb-doping of the Ti_3_C_2_T_x_ increased interlayer spacing and O-group concentration and improved ion diffusion and surface reactivity. Shen et al. [49] presented the development of flexible SCs by employing OH-WCNT/Nb_2_CTx MXene conductive sponge-supported PAN nanofiber membranes. The authors found that the MX_0.5_CNT_0.5_ electrode exhibits a Csp of 8 F/cm^3^ at 0.5 A/cm^3^ and maintains 92.2% capacitance after 10,000 cycles. Cai et al. [50] also proposed a novel 3D-MXene-unity membrane aerogel for flexible SCs, which showed a C_sp_ of 215 F/g. Lu et al. [51] explored cobalt (Co) ion chelated strategies for the preparation of Co@Ti_3_C_2_T_x_ MXene for SCs. The preparation of the Co@Ti_3_C_2_T_x_ is shown in Figure 2a. The first step involves an etching process to prepare Ti_3_C_2_T_x_ from the Ti_3_AlC_2_ MAX phase. Furthermore, an alkalizing process was carried out to obtain the alkalizing Ti_3_C_2_T_x_ MXene. The Co^2+^ ions were introduced using a chelating process, which yielded Co@Ti_3_C_2_T_x_ MXene. The XRD patterns of the various materials, including Ti_3_AlC_2_ MAX, Ti_3_C_2_T_x_ MXene, alkalizing Ti_3_C_2_T_x_ and 1-Co@Ti_3_C_2_T_x_, are displayed in Figure 2b. The XRD studies confirmed the formation of the desired materials with acceptable phase purity. The Raman studies also authenticated the formation of Ti_3_C_2_T_x_ MXene and 1-Co@Ti_3_C_2_T_x_, as shown in Figure 2c. The authors also employed XPS studies to analyze the elemental and oxidation states of Ti_3_C_2_T_x_ MXene and 1-Co@Ti_3_C_2_T_x_. The XPS results are shown in Figure 2d–h. The survey spectrum revealed the presence of C1s, Ti2p, O1a and F1s for Ti_3_C_2_T_x_ MXene, whereas C1s, Ti2p, O1s and Co2p were observed in the XPS survey spectrum of 1-Co@Ti_3_C_2_T_x_ (Figure 2d). The core XPS spectra of the Ti2p, C1s, O1s and Co2p are displayed in Figure 2e, Figure 2f, Figure 2g and Figure 2h, respectively. The authors found that desired materials were formed with decent phase purity and acceptable crystallinity. The prepared Ti_3_C_2_T_x_, 0.5-Co@Ti_3_C_2_T_x_, 1-Co@Ti_3_C_2_T_x_ and 2-Co@Ti_3_C_2_T_x_ electrode materials were explored for SCs. The GCD studies for Ti_3_C_2_T_x_ show a higher Csp value at 1 A/g (Figure 2i). Similarly, GCD data for 0.5-Co@Ti_3_C_2_T_x_, 1-Co@Ti_3_C_2_T_x_ and 2-Co@Ti_3_C_2_T_x_ exhibited a higher Csp value at 1 A/g, as shown in Figure 2j, Figure 2k and Figure 2l, respectively. The relationship between the specific capacity and the current density of the various electrode materials is displayed in Figure 2m. The optimized 1-Co@Ti_3_C_2_T_x_ MXene exhibited a higher C_sp_ of 48 mAh/g at 1 A/g. This study suggested the potential of chelating agents for the construction of energy storage materials.

Cui et al. [52] presented the use of MXene/biomass/chitosan (Cs) carbon aerogel (MBC) as a capacitor material and reported a Csp of 1801.4 mF/cm^3^ at 2 mV/s under optimized conditions. Zhao et al. [53] reported a Csp of 210.1 mF/cm^2^ with excellent cyclic stability with 10,000 cycles using hexaazatriphenylene-doped MXene, whereas Yan et al. [54] reported a Csp value of 223.4 F/g at 1000 mV/s with 10,000 cycles for porous MXene-based SCs. These reports showed that MXene has excellent electrochemical properties for SCs. Furthermore, Kumar et al. [55] also used novel approaches to enhance the properties of the Ti_3_C_2_T_x_ MXene, and electrochemical studies showed a Csp of 321 F/g at 1 A/g. Another study by Wan et al. [56] involves the formation of MXene by employing three amino sources, i.e., EDA, MEA and HM, to enhance the energy storage properties of the MXene-based SCs. The effects of the EDA, MEA and HM on the surface morphology of the Ti_3_C_2_ were examined by using the FESEM method.

**Figure 3:**
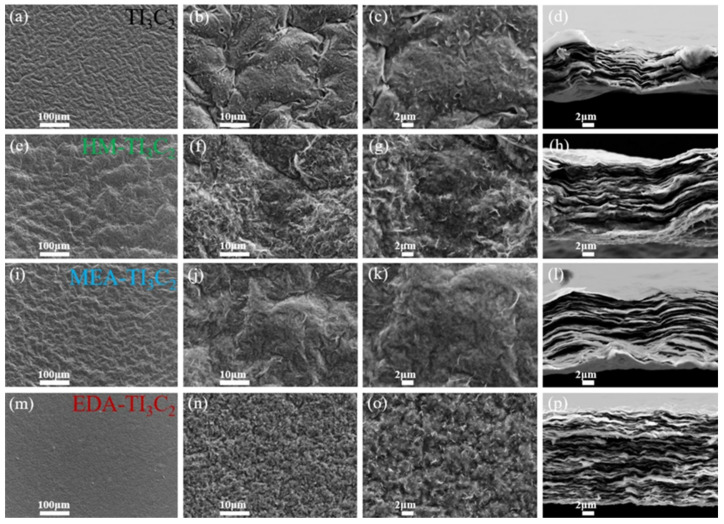
Top-view (**a**–**c**) and cross-sectional (**d**) FESEM images of Ti_3_C_2_. Top-view FESEM pictures (**e**–**g**) and cross-sectional (**h**) images of HM-Ti_3_C_2._ Top-view FESEM pictures (**i**–**k**) and cross-sectional (**l**) images of MEA-Ti_3_C_2._ Top-view FESEM pictures (**m**–**o**) and cross-sectional (**p**) images of EDA-Ti_3_C_2._ Reproduced with permission from reference [56].

The FESEM images of the top and cross-sectional views for the Ti_3_C_2_ sample are shown in Figure 3a–c and Figure 3d, respectively. Similarly, top and cross-sectional views of the FESEM images of the HM-Ti_3_C_2_ are displayed in Figure 3e–g and Figure 3h, respectively. Figure 3i–k and Figure 3l show the top view and cross-sectional view of the surface morphology of the prepared MEA-Ti_3_C_2_ sample, respectively. The FESEM images of the top and cross-sectional surface morphology of the EDA-Ti_3_C_2_ are depicted in Figure 3m–o and Figure 3p, respectively. The morphological studies exhibited the presence of a similar 2D layered structure and interlayer stacking in the prepared samples. However, the degree of stacking was found to be alleviated for HM-Ti_3_C_2_, MEA-Ti_3_C_2_ and EDA-Ti_3_C_2_ samples compared to the Ti_3_C_2_. It was observed that the surface of the EDA-Ti_3_C_2_ sample exhibits a more uniform and delicate texture compared to the other samples. Moreover, the interlayer spacing in the EDA-Ti_3_C_2_ sample was enlarged compared to the HM-Ti_3_C_2_ and MEA-Ti_3_C_2_ samples. In addition, EDA-Ti_3_C_2_ displayed cleanliness without the presence of wrinkled skin and protrusions of Ti_3_C_2_ (Figure 3p). Thus, EDA-modified MXene exhibited a Csp of 683 F/g in 1 M sulfuric acid (H_2_SO_4_) electrolyte with 10,000 cycles. The presence of amino groups improved the electrochemical properties of the MXene, and enhanced Csp was observed for EDA-modified MXene-based SCs. Liang et al. [57] prepared Ti_3_C_2_T*_x_*/ammonium polyphosphate (APP-A-3h) for the construction of SCs and obtained a Csp of 597.8 F/g, while pristine MXene exhibited a Csp of 384 F/g. Cai et al. [58] reported that the preparation of magnesium (Mg^2+^) induced Ti_3_C_2_T_x_ MXene@microfibrillated cellulose aerogels supported by a melamine sponge (Mg-MFMX@MS) under benign conditions. This work demonstrates that the Mg-10 %MFMX@MS aerogel achieves a high area capacitance (685.77 mF/cm^2^ at 10 mV/s) with 10,000 cycles. It was observed that the use of Mg^2+^ as a cross-linking agent may significantly prevent the restacking of MXene nanosheets by improving interlayer spacing. Rafique et al. [59] utilized 2D molybdenum nitride (Mo_2_N) MXene for SCs. The authors prepared 2D Mo_2_N using a solid-state reduction–nitridation method. The electrochemical studies showed a Csp of 1272.45 F/g (10 mV/s). This may be due to the presence of a larger surface area, porosity and active sites for electrochemical reactions.

### 3.2. MXenes/Metal Oxides Based SCs

**Figure 4:**
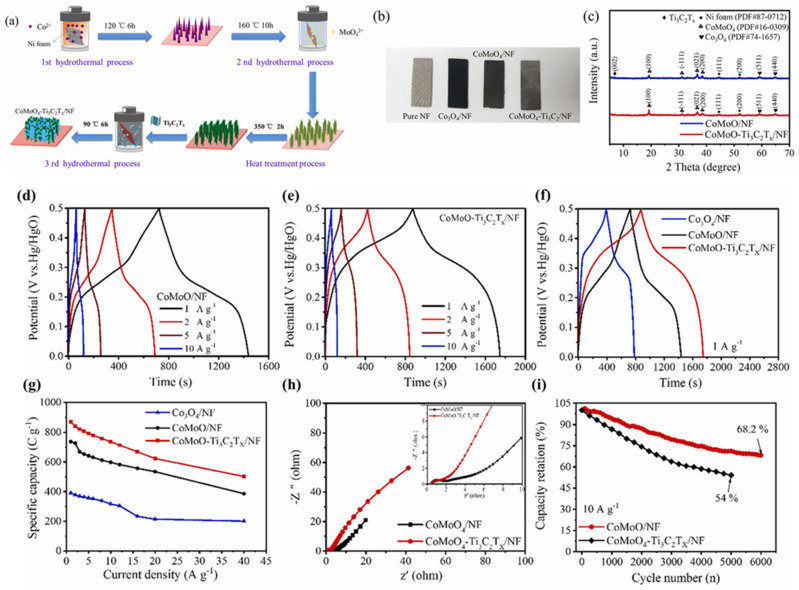
(**a**) Schematic picture of the preparation of CoMoO_4_-Ti_3_C_2_T_x_/NF. (**b**) Digital picture of the various prepared electrodes. (**c**) XRD patterns of the prepared samples. GCD curves of the (**d**) CoMoO_4_/NF and (**e**) CoMoO_4_-Ti_3_C_2_T_x_/NF at different current densities. GCD curves of (**f**) Co_3_O_4_/NF, CoMoO_4_/NF and CoMoO_4_-Ti_3_C_2_T_x_/NF at 1 A/g. (**g**) Specific capacity versus current density relations of various electrodes. (**h**) Nyquist plots of CoMoO_4_/NF and CoMoO_4_-Ti_3_C_2_T_x_/NF. (**i**) Cyclic stability results of CoMoO_4_/NF and CoMoO_4_-Ti_3_C_2_T_x_/NF. Reproduced with permission from reference [63].

Zhang et al. [60] proposed the synthesis of Cu_1.5_Mn_1.5_O_4_ hollow nanosphere (HNS)-modified Ti_3_C_2_T_x_ MXene (CuMnHS@MX) composites and examined their electrochemical properties for SCs. The CuMnHS@MX hybrid film, prepared via electrostatic self-assembly and vacuum filtration, exhibits remarkable electrochemical properties, achieving a high Csp of 2089 mF/cm^2^ at 2.5 mA/cm^2^ with stability at 8000 cycles. It was proposed that the improved performance may be due to the 3D structure of the proposed composite, which may provide increased interlayer spacing and enhanced ion transport. Vetrikarasan et al. [61] employed a one-step synthesis method for the preparation of λ-MnO_2_ nanoplates and combined it with 2D Ti_3_C_2_T_x_ MXene. The λ-MnO_2_//Ti_3_C_2_T_x_ SCs exhibited excellent cyclic stability with 5000 cycles. The presence of the synergism between λ-MnO_2_ and Ti_3_C_2_Tx enhances the capacitive properties for SCs. Ghaemi et al. [62] prepared Ni_0.5_Zn_0.5_Fe_2_O_4_/Ti_3_C_2_T_x_ MXene using simple strategies, and its electrochemical properties were checked for SCs. The Ni_0.5_Zn_0.5_Fe_2_O_4_-25 wt %/Ti_3_C_2_T_x_ MXene exhibited a Csp of 1614 C/g at 1 A/g. Wei et al. [63] reported the synthesis of cobalt molybdate (CoMoO_4_)-Ti_3_C_2_T_x_ MXene composites by using the hydrothermal method, as shown in Figure 4a. The nickel foam (NF) was used as an electrode substrate for the preparation of CoMoO_4_ arrays/Ti_3_C_2_T_x_ MXene composites. The digital pictures of the pure NF, Co_3_O_4_/NF, CoMoO_4_/NF and CoMoO_4_-Ti_3_C_2_T_x_/NF are displayed in Figure 4b. The XRD studies confirmed the formation of CoMoO_4_-NF and CoMoO_4_-Ti_3_C_2_T_x_/NF with acceptable phase purity (Figure 4c).

**Figure 5:**
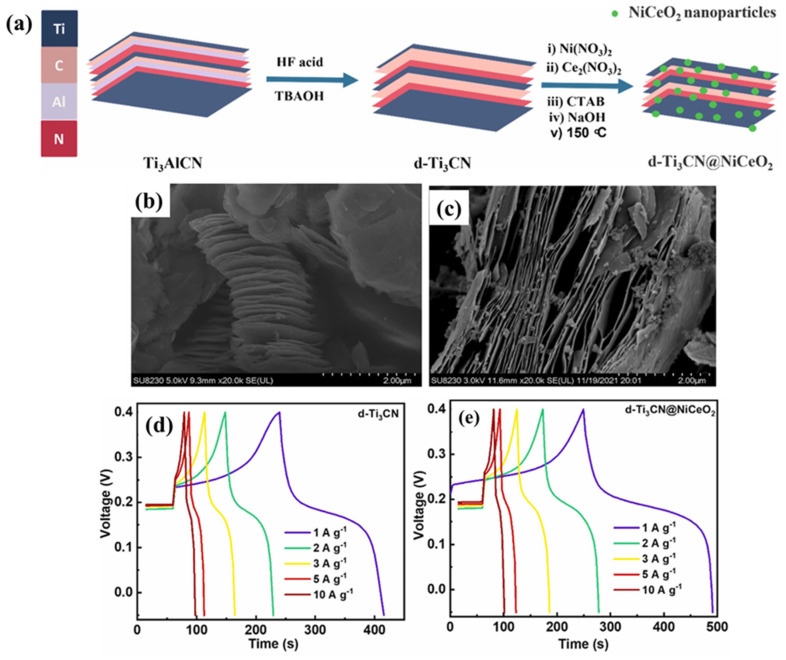
(**a**) Schematic representation of the formation of d-Ti_3_CN@NiCeO_2_. SEM image of (**b**) d-Ti_3_CN and (**c**) d-Ti_3_CN@NiCeO_2_. Scale bar is 2 µm. GCD curves of (**d**) d-Ti_3_CN and (**e**) d-Ti_3_CN@NiCeO_2_ at various current densities. Reproduced with permission from reference [69].

The authors employed the synthesized CoMoO_4_/NF and CoMoO_4_-Ti_3_C_2_T_x_/NF as electrodes for the development of SCs. The GCD curves of the CoMoO_4_/NF and CoMoO_4_-Ti_3_C_2_T_x_/NF at various current densities are depicted in Figure 4d and Figure 4e, respectively. It can be observed from the GCD data that CoMoO_4_/NF shows a decent Csp value at 1 A/g, but CoMoO_4_-Ti_3_C_2_T_x_/NF exhibits a further improved Csp value at 1 A/g. Thus, it is clear that the presence of synergistic interactions between the CoMoO_4_ and Ti_3_C_2_T_x_ enhanced the electrochemical performance of the CoMoO_4_-Ti_3_C_2_T_x_/NF compared to the CoMoO_4_/NF- and Co_3_O_4_/NF-based SCs (Figure 4f). The specific capacity versus current density graph of the Co_3_O_4_/NF, CoMoO_4_/NF and CoMoO_4_-Ti_3_C_2_T_x_/NF is shown in Figure 4g. The authors achieved a decent Csp of 870.7 C/g (1 A/g). The Nyquist plots of the CoMoO_4_/NF and CoMoO_4_-Ti_3_C_2_T_x_/NF are summarized in Figure 4h and indicate that CoMoO_4_/NF and CoMoO_4_-Ti_3_C_2_T_x_/NF have series resistances (R_s_) of 0.45 and 0.40 Ω, respectively. The authors stated that CoMoO_4_/NF and CoMoO_4_-Ti_3_C_2_T_x_/NF did not show a semicircle shape, and it was assumed that the charge transfer resistance (R_ct_) value is smaller. It was also found that the optimized electrode has a retention of 68.2% cyclic stability after 6000 cycles at 10 A/g (Figure 4i). Pathak et al. [64] also explored the potential of MnCo_2_O_4_/MWCNT/Ti_3_C_2_T_x_ (MCO/TCX) composites for SC applications and obtained a Csp of 860.22 F/g (2 A/g) with excellent stability at 5000 cycles. Wang et al. [65] obtained a MOF-derived hollow Co_9_S_8_ core@multi-shell structure and combined it with MXene and Bi_2_O_3_. The fabricated CS-2@MXene@Bi_2_O_3_ was used as SC material, which showed a Csp of 646.1 F/g (1 A/g). The unique structure consists of MXene flakes and Bi_2_O_3_ nanosheets, showing enhanced conductivity and electro-active sites, which increase the Csp of the proposed SCs. In another study, Luo et al. [66] obtained a Csp of 348.5 F/g (0.5 A/g) with excellent stability at 5000 cycles using MnO_2_ nanoflowers/MXene (MNF/Ti_3_C_2_Tx). Luo et al. [67] found that flexible SCs can be fabricated by using Ti_3_C_2_T_x_ MXene/V_2_O_5_ (MV) electrode material. This proposed study demonstrated decent electrocatalytic and capacitive properties of the proposed material, and the authors reported a Csp of 319.1 F/g at 0.5 A/g with 5000 stability cycles. Karmur et al. [68] also discussed the electrochemical properties of the MXene/WO_3_@rGO sponge (sp) for the construction of flexible SCs. The authors were able to achieve an excellent Csp of 774.4 F/g at 5 A/g and an energy density of 34 Wh/kg at a power density of 1450 W/kg with 3000 stability cycles. Ashraf et al. [69] explained the preparation of novel d-Ti_3_CN@NiCeO_2_ using the hydrothermal method (Figure 5a). In the first step, d-Ti_3_CN MXene was prepared by employing the molten salt method and the hydrofluoric acid (HF) etching process. Furthermore, the hydrothermal method was used for the preparation of d-Ti_3_CN@NiCeO_2_.

**Figure 6:**
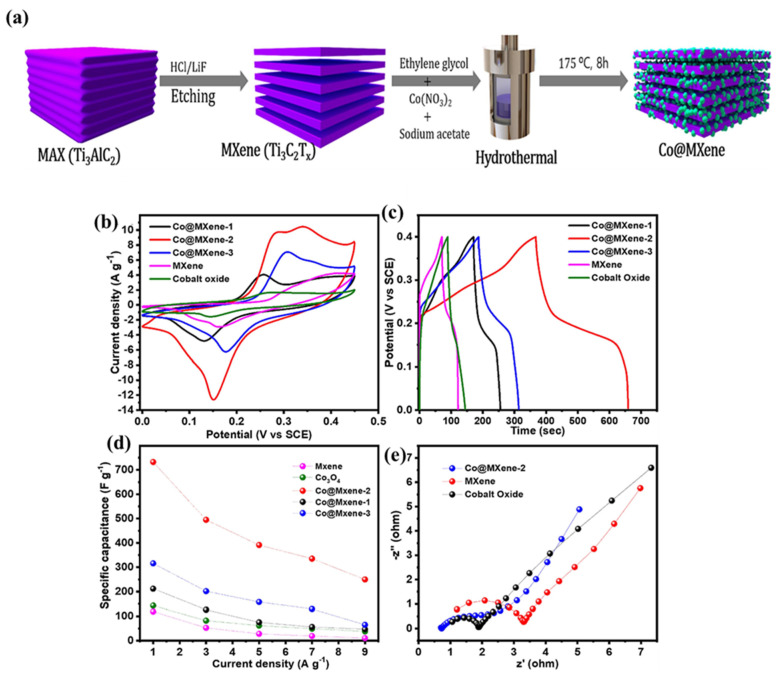
(**a**) Schematic representation of the preparation of Co@MXene composite. (**b**) CVs and (**c**) GCD curves of different materials. (**d**) Specific capacitance–current density relation of different materials. (**e**) Nyquist plots of different electrode materials. Reproduced with permission from reference [76].

The SEM image of the d-Ti_3_CN and d-Ti_3_CN@NiCeO_2_ is displayed in Figure 5b and Figure 5c, respectively. The GCD curves of the d-Ti_3_CN and d-Ti_3_CN@NiCeO_2_ at varied current densities are depicted in Figure 5d and Figure 5e, respectively. It was observed that d-Ti_3_CN synthesized with the molten salt method has better electrochemical activity. The highest Csp of 941 F/g was obtained for d-Ti_3_CN@NiCeO_2-_based SCs at 1 A/g under a 2 M KOH system. This enhanced electrochemical activity may be explained on the basis of synergistic interactions between d-Ti_3_CN MXene and NiCeO_2_, which provide high conductivity and increase active sites and capacitance. Beknalkar et al. [70] adopted a CuMn_2_O_4_/Ti_3_C_2_ MXene composite as an electrode material for SCs and observed that the 3 h deposition-based sample has excellent electrochemical properties and enhanced electrical conductivity. Thus, the proposed material demonstrated a Csp of 628 mF/cm^2^ at 4 mA/cm^2^. According to Reina et al. [71], a Csp of 165.5 mF/cm^2^ can be obtained by using laser-induced graphene (LIG)/MXene/manganese oxide composites. This study showed the potential of LIG/MXene-based composites for flexible SC applications. Althubiti et al. [72] presented the synthesis of a MnFe_2_O_4_/MXene for SC applications, and electrochemical studies showed a Csp of 1263.01 F/g (1 A/g). Nikhil et al. [73] employed three synthesis methods (in situ delamination, solvothermal synthesis and bath sonication) for the preparation of Ti_3_C_2_T_x_ MXene for SCs. The synthesized MXene was combined with NiO, and the optimized results showed a Csp of 770 C/g (1 A/g). Ammar et al. [74] obtained Mn-doped ZnO and Cu-doped ZnO using the hydrothermal method and the co-precipitation method, respectively. The synthesized Mn-doped ZnO and Cu-doped ZnO were combined with MXene, and the authors observed that 2% Mn-doped ZnO possesses better electrochemical properties, and the optimized results demonstrated a Csp of 151F/g. Sun et al. [75] proposed the synthesis of a MXene/TiO_2_-graphene aerogel (MXene/TiO_2_-Fe-G) for ionic liquid-based SCs. The authors found that the 3D structure of the prepared composite and the presence of amorphous nature of the TiO_2_ on the MXene surface provide a larger surface area for electrochemical reactions. Thus, a Csp of 196.4 F/g was obtained at 1 A/g for MXene/TiO_2_-Fe-G-based SCs. Kunwar et al. [76] reported the synthesis of cobalt oxide (Co_3_O_4_) NPs/MXene nanocomposites (Co@MXene) for high-performance SCs. The fabrication of the Co@MXene has been explained in Figure 6a. The authors prepared various materials to optimize their electrochemical properties for SCs. The CVs of the MXene, Co_3_O_4_, Co@MXene-1, Co@MXene-2 and Co@MXene-3 are shown in Figure 6b. The studies showed that Co@MXene-2 is a highly efficient capacitive material compared to the other materials. The GCD of the MXene, Co_3_O_4_, Co@MXene-1, Co@MXene-2 and Co@MXene-3 are demonstrated in Figure 6c. The GCD results clearly show that MXene and Co_3_O_4_ have lower electrochemical activity, whereas Co@MXene-2 has the highest electrochemical activity for SCs (Figure 6d). Thus, the Co@MXene-2 composite-based electrode exhibited a Csp of 732.5 F/g (1 A/g). This is attributed to the higher electrical conductivity of Co@MXene-2 compared to the other electrode, as suggested by EIS studies (Figure 6e). The appearance of a low Rct value for Co@MXene-2 may be due to the presence of synergistic interactions between highly conductive MXene and Co_3_O_4_. The existence of synergism may provide an efficient charge transfer pathway, improve the electron transport between the layers of MXene and decrease the resistance to charge transfer. The proposed material also suggested the presence of 5000 stability cycles. Bin et al. [77] fabricated free-standing delaminated vanadium carbide MXene (d-V_4_C_3_T_x_)/molybdenum trioxide (MoO_3_) for SCs, achieving a Csp of 645 C/g at 1 A/g with stability at 10,000 cycles. Prabhakar et al. [78] presented the synthesis of Nb_4_C_3_T_x_ MXene/WO_3_ for SCs, achieving a high Csp of 1045 F/g (1 A/g) and stability at 5000 cycles. Wang et al. [79] obtained a Csp of 1025 F/g (1 A/g) for Ti_3_C_2_T_x_ MXene/NiCo_2_O_4_ nanosphere composites-based SCs. This prepared electrode material was also stable up to 5000 cycles.

Xu et al. [80] employed g-C_3_N_4_/MoO_3_-Ti_3_C_2_T_x_ MXene (CMM) as an energy storage material and obtained a Csp of 1168 F/g (1 A/g) in 1 M H_2_SO_4_ with stability at 5000 cycles. Li et al. [81] achieved a Csp of 1786 F/g at 1 A/g with stability at 10,000 cycles using NiCo_2_S_4_MXene/N-doped carbon (NiCo_2_S_4_/MXene/NC) as the electrode material. Shahid et al. [82] reported the formation of the CeO_2_/MXene/PANI composite using hydrothermal and in situ polymerization methods. The authors reported the preparation of CeO_2_/MXene, resulting in three ternary composites, namely (CeO_2_/MXene)/PANI 80%:20% (CMP1), (CeO_2_/MXene)/PANI 60%:40% (CMP2) and (CeO_2_/MXene)/PANI 40%:60% (CMP3), for SCs. The CMP3 electrode showed better electrochemical performance for SCs, and the authors were able to obtain a Csp of 2247.962 F/g with stability at 6000 cycles. Chen et al. [83] proposed the formation of CuCo_2_O_4_-HS composite and found that the synthesized material has hollow spherical-shaped (HS) microspheres. The synthesized material was combined with MXene to form the CuCo_2_O_4_-HS/MXene for SCs. The electrochemical studies showed that a Csp of 1341.4 C/g can be obtained at 1 A/g. Li et al. [84] employed the hydrothermal method for the synthesis of MXene/NiCo_2_S_4_ (MNCS) and observed that the synthesized NiCo_2_S_4_ was uniformly distributed on the surface or within the interlayers of MXene. The authors obtained a Csp of 2675 F/g at 1 A/g with 10,000 stability cycles in a three-electrode system. Ikram et al. [85] reported the construction of MoO_3_/MnFe_2_O_4_/MXene-based electrodes for SCs, which exhibited a Csp of 817 F/g with 10,000 stability cycles. Mathew et al. [86] also designed and fabricated MXene/cobalt ferrite (CoFe_2_O_4_)/graphitic carbon nitride (g-C_3_N_4_) composites (MCG) for SC applications. The authors also prepared MXene/CoFe_2_O_4_ (MC) as a control sample.

**Figure 7:**
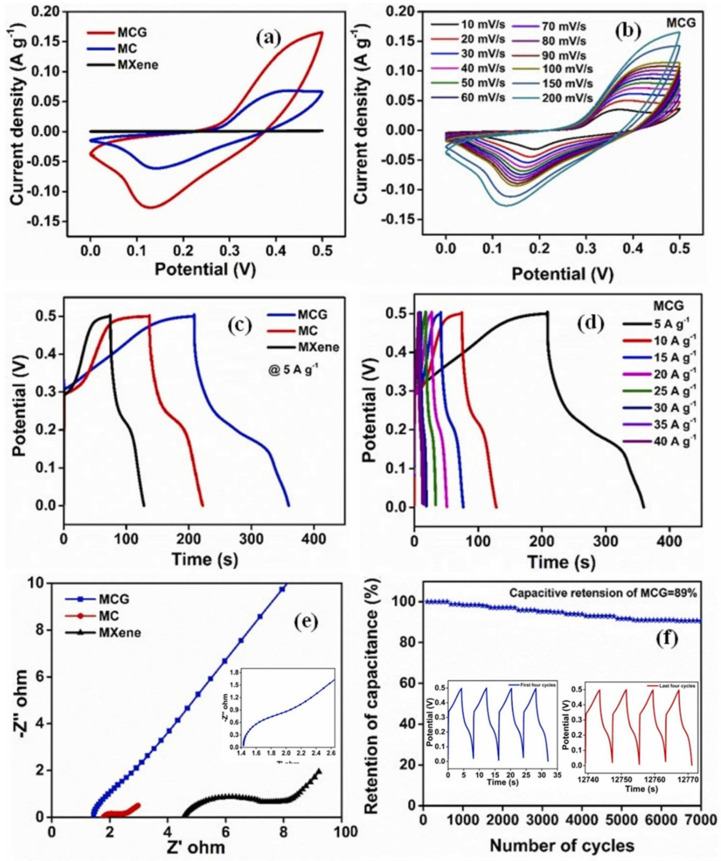
(**a**) CVs of the MC, MCG and MXene at 200 mVs^−1^. (**b**) CV curves of MCG at different scan rates. (**c**) GCD curves of MC, MCG and MXene at 5 A/g. (**d**) GCD curves of MCG at 5 to 40 A/g. (**e**) Nyquist plots of MC, MCG and MXene (inset shows enlarged plot for MCG). (**f**) Stability data. Reproduced with permission from reference [86].

The CVs of the MC, MXene and MCG samples are displayed in Figure 7a, revealing that MXene has the worst electrochemical properties, whereas MCG has the highest electrochemical activity for SCs. The CVs of the MCG at various adopted scan rates are displayed in Figure 7b. The authors found that the MCG sample shows a low capacitance value at a higher scan rate but a higher capacitance value at a lower scan rate. It was stated that at a low scan rate, electrolyte ions have ample time to diffuse into the interior components of the electrode and may interact with more active sites. Thus, enhanced capacitance can be observed at low scan rates. Figure 7c displays the reported GCD data for the proposed MCG-, MC- and MXene-based SCs. MCG exhibited a higher Csp value compared to the MC and MXene-based SCs. Similarly, Figure 7d depicts the reported GCD data of the MCG sample under various current densities. It was found that the highest Csp of 1506.2 F/g can be obtained at 5 A/g in a 3 M KOH system. The EIS results for the MXene, MC and MCG are shown in Figure 7e, revealing that the MCG sample has acceptable electrical conductivity. The stability data for MCG-based SCs is given in Figure 7f. The observations revealed that MCG has acceptable stability at 7000 cycles. Akbar et al. [87] prepared MgCo_2_O_4_ (MC) and Ag-MgCo_2_O_4_ (AMC) by the hydrothermal method. Further, Ag-MgCo_2_O_4_ (AMC) was coupled with MXene sheets by the ultrasonication method to obtain the AMC/MXene composite. The AMC/MXene showed a Csp of 1722 F/g (1 A/g). Khan et al. [88] proposed the synthesis of NiCo_2_O_4_@MXene composite (NCO@MXene) and employed it as SC material. The authors obtained a Csp of 777.7 F/g (1 A/g) with 10,000 stability cycles. Verma et al. [89] synthesized MXene/NiO (MX/NiO) using the bath sonication method and employed it as an electrode material for the development of SCs, which yielded a Csp of 892 F/g at 1 A/g, which is higher than pristine MXene-based SCs (358.5 F/g), whereas Meenakshi et al. [90] obtained a Csp of 500 F/g (1 A/g) for CuCoTiO_2_/MXene-based SCs.

### 3.3. MXenes/Metal Sulfides/Selenides Based SCs

**Figure 8:**
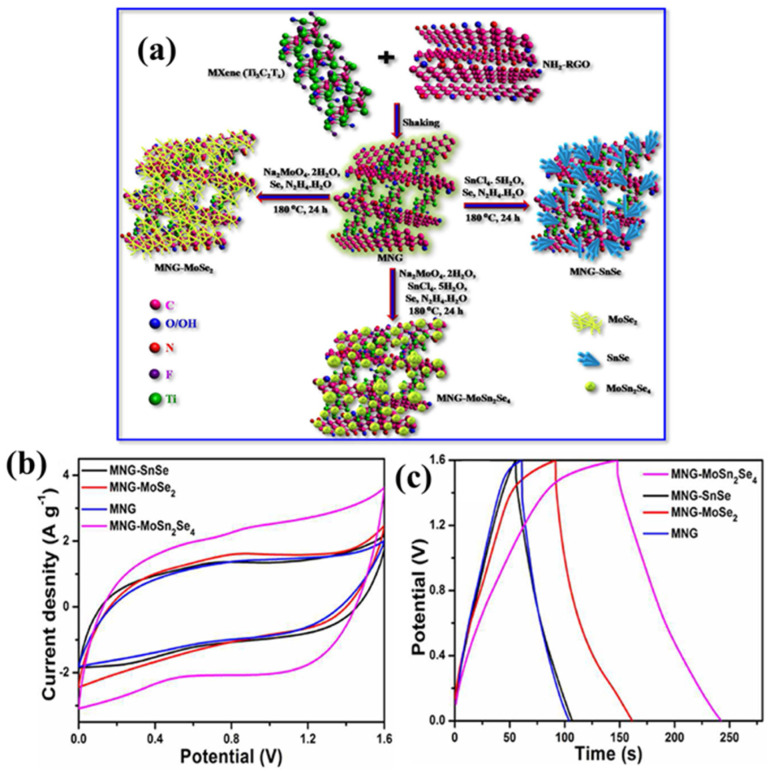
(**a**) Schematic illustration of the synthesis of MNG, MNG-MoSe_2_, MNG-SnSe and MNG-MnSn_2_Se_4_. (**b**) CV and (**c**) GCD curves of the different electrode materials. Reproduced with permission from reference [95].

Hussain et al. [91] synthesized WS_2_@MXene/GO and obtained a Csp of 1111 F/g (2 A/g), whereas Li et al. [92] utilized C-Ti_3_C_2_T_x_/CuS as electrode material, which yielded a Csp of 1186 F/g (1 A/g). Chen et al. [93] prepared a MXene/VS_2_ composite and employed it as an SC material and obtained a Csp of 1791.4 F/g at 1 A/g with 10,000 stability cycles. De et al. [94] used the hydrothermal method for the formation of tin selenide (SnSe_2_) and combined it with activated porous carbon (APC)/Ti_3_C_2_T_x_ (MXene) for the development of SCs. The authors found that APC/Ti_3_C_2_T_x_/SnSe_2_ exhibited a Csp of 815 F/g in a three-electrode system. De et al. [95] also explored MoSn_2_Se_4_-decorated MXene/functionalized rGO as an electrode material for SCs. The synthetic procedures for MNG, MNG-MoSe_2_, MNG-SnSe and MNG-MnSn_2_Se_4_. (MNG = MXene and NH_2_-RGO) are shown in Figure 8a. The CV curves of MNG, MNG-MoSe_2_, MNG-SnSe and MNG-MnSn_2_Se_4_ are displayed in Figure 8b. It is clear that MNG-MnSn_2_Se_4_ has better electrochemical properties compared to the other materials. The GCD results of MNG, MNG-MoSe_2_, MNG-SnSe and MNG-MnSn_2_Se_4_ are presented in Figure 8c. The obtained GCD data shows that a Csp of 120.2 F/g was observed using MNG-MnSn_2_Se_4_.

Adil et al. [96] prepared MXene-cobalt sulfide (CoS) for the development of SCs and reported a Csp of 447 mAh/g at 3 mA/cm^2^. In another study [97], CoS/MXene/PANI and CoS/MXene/PEDOT composites were reported for the construction of SCs. The authors found that CoS/MXene/PANI- and CoS/MXene/PEDOT-based electrodes exhibit a Csp of 246 F/g and 331.1 F/g, respectively. Zhang et al. [98] obtained a Csp of 491 F/g (1 A/g) using a flower-like CuS-modified MXene nanocomposite as the electrode material. Chen et al. [99] employed a Ti_3_C_2_T_x_ MXene/CuS composite as the electrode material and achieved a Csp of 2569.3 F/g (1 A/g) with excellent stability at 10,000 cycles. Sun et al. [100] reported the construction of NiS/N-MXene for SC applications. This electrode material has a Csp of 429 F/cm^3^ and an energy density of 33.5 mWh/cm^3^. Arulkumar et al. [101] proposed the formation of Ti_3_C_2_T_x_ MXene/MoSe_2_ composites and achieved a Csp of 1531.2 F/g at 1 A/g. Kim et al. [102] used novel strategies for the development of Sn-Co-S/MXene composites for SC applications and achieved a Csp of 305.71 mAh/gm (1 A/g) with 10000 stability cycles. Qiao et al. [103] also utilized MXene/1T-MoS_2_ as an electrode material, which exhibited decent stability and electrochemical performance. Ranjith et al. [104] reported the fabrication of CoSe_2_ (MXe-F, N-*g*CW-CoSe_2_) for SC applications and obtained a Csp of 403.4 C/g (1 A/g) with 10,000 stability cycles. As per Xiao et al. [105], a Csp of 1221.6 F/g can be obtained for CoNi_2_S_4_/carbon/MXene-based SCs with excellent stability at 30,000 cycles. Hayat et al. [106] found that MoS_2_@MXene//MXene flexible SCs device offers a decent energy density of 1.21 W h/kg and a power density of 54.45 W/kg. Liang et al. [107] used MoS_2_/MXene as an electrode material and obtained an areal capacitance of 0.91 mAh/cm^2^ at 2 mA/cm^2^. Kumar et al. [108] reported the construction of MXene/FeNi_2_S_4_ composite-based electrodes for SC application and obtained a Csp of 673 F/g (1 A/g). Chen et al. [109] obtained a Csp of 702.7 C/g (1 A/g) for MXene/graphene/CoNiSe (MG-CoNiSe)-based SCs. Dey et al. [110] explored the energy storage properties of NiSe/MXene and obtained a Csp of 65.4 mAh/cm^3^ at 0.4 mA, whereas Pinar et al. [111] obtained a Csp of 373 F/g at 0.4 A/g using MXene/WS_2_-based electrodes.

### 3.4. MXenes/Polymers Based SCs

**Figure 9:**
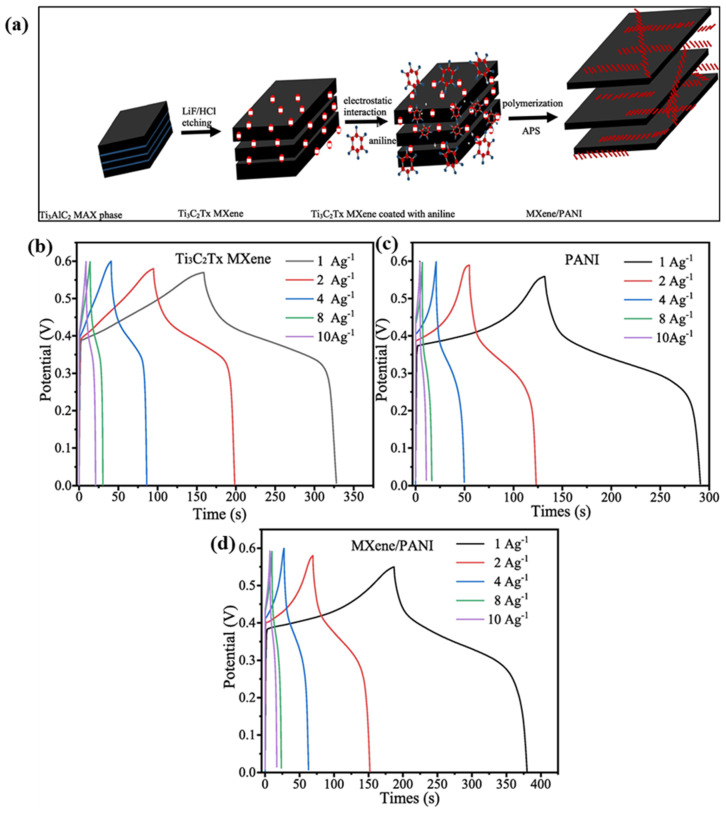
(**a**) Schematic graph of the formation of MXene/PANI. GCD curves of (**b**) Ti_3_C_2_T_x_ MXene, (**c**) PANI and (**d**) MXene/PANI at different current densities. Reproduced with permission from reference [116].

Zheng et al. [112] explored the potential of poly(vinyl alcohol) (PVA)/bacterial cellulose (BC)/MXene (PBM) for SC applications. It was found that a Csp of 3948 mF/cm^2^ at 0.47 mA/cm^2^ can be obtained for PPy@PVA/BC/MXene (PPy@PBM)-based SCs. Another report by Lin et al. [113] highlighted the role of CC/MXene/PANI/CoNi-LDH (CC/MXPACN) composites as an energy storage material and found that the proposed material has 10000 stability cycles with a power density of 399.95 W/kg and an energy density of 39.33 Wh/kg. In another study [114], MXene/polypyrrole (M-PPy) was also used as an electrode material for SCs, and the authors obtained a Csp of 563.8 F/g (0.5 A/g) for -PPy3 (3 mL PPy)-based SCs. Yuan et al. [115] also used Ti_3_C_2_T_x_/CNF/PANI composites for SC applications and achieved a Csp of 2935 mF/cm^2^ (1 mA/cm^2^) under optimized conditions. Chen et al. [116] adopted novel strategies for the preparation of Ti_3_C_2_T*_x_*/PANI composites for energy storage applications. The schematic picture for the preparation of Ti_3_C_2_T*_x_*/PANI is shown in Figure 9a. The synthesized material was characterized by various methods and employed as an electrode modifier for SCs. The GCD data of the Ti_3_C_2_T_x_ MXene, PANI and MXene/PANI at different current densities are shown in Figure 9b, Figure 9c and Figure 9d, respectively. The electrochemical studies showed that PANI-based SCs exhibited a lower Csp, whereas an improved Csp was observed for MXene-based SCs. However, the highest Csp of 458.3 F/g was observed (5 mV/s) for MXene/PANI-based SCs.

In another study [117], MXene-integrated hollow carbon nanofibers (MXHCNFs) were prepared via co-axial electrospinning, and the inside and outside of MXHCNFs were decorated with Ppy layers to form the PPy@MXHCNF. Furthermore, ZnCoMOF was grown on the above-prepared material and explored as a precursor for the preparation of ZCO@PPy@MXHCNF as a positive electrode. The NPC@MXHCNF was used as a negative electrode, and the developed SCs (ZCO@PPy@MXHCNF//NPC@MXHCNF) showed an energy density of 61.3 Wh/kg at a power density of 796.8 W/kg. The authors also reported that free-standing positive and negative electrodes demonstrated a Csp of 1567.5 F/g and 477.2 F/g at 1 A/g, respectively. Wang et al. [118] proposed the formation of aramid nanofiber-reinforced (ANF) MXene/PEDOT:PSS hybrid fibers using a simple and tractable wet spinning strategy. This study showed a high energy density of 9.8 mWh/cm^3^ at a power density of 250.7 mW/cm^3^. Xie et al. [119] synthesized PANI/MoO_x_/MXene (PMM) for its applications in SCs. It was found that the 20 wt % PANI/MoOx-based electrode (PMM-20) has a Csp of 450 F/g at 5 mV/s. Xiang et al. [120] also explored PANI/MXene composites for the development of SCs and reported a Csp of 256.7 mF/cm, whereas Bai et al. [121] obtained a Csp of 222 F/g using PANI/MXene composite-based SCs. Fei et al. [122] proposed the preparation of SCs using a PANI-deposited bacterial cellulose (BC) membrane and an MXene/CNTs film. The MXene/CNT-12%-based SCs showed a Csp of 198 F/g. Mohammadi et al. [123] used Ti_3_C_2_T_x_ MXene/PANI/polyvinylidene fluoride (PVDF) as an energy storage material and achieved a Csp of 740 F/g (2 mV/s) and 895 F/g (0.5 A/g). Lima et al. [124] employed dodecylbenzenesulfonic acid (DBSA)-doped PANI and PPy as effective spacers and additional pseudocapacitance agents for Ti_3_C_2_T_x_ MXene. The observations showed that a Csp of 270 F/g can be obtained at 1 A/g by adding 10 wt % PPy. Bai et al. [125] also reported the fabrication of MXene/g-PPy@sulfonated cellulose composite electrodes for SCs, which exhibited excellent stability at 10,000 cycles. Khan et al. [126] prepared ternary composites consisting of PPy, Mxene and Gum Arabic (PPy/Mxene/GA), and the PPy/Mxene/GA-based electrode demonstrated a Csp of 657.64 F/g (1 A/g). Han et al. [127] obtained a Csp of 847 mF/cm^2^ for MXene/PANI-based SCs, whereas Ma et al. [128] achieved a Csp of 345 F/g with stability at 5000 cycles for PEDOT:PSS/MXene/PPy-based SCs. Ma et al. [129] designed and prepared sulfonated polyaniline/MXene for SC applications, which yielded a Csp of 512.45 F/g at 1 A/g. Bejjanki et al. [130] utilized PANI/WO_3_/MXene as an electrode material, which revealed that a Csp of 741 F/g can be achieved at 1 A/g. In another study [131], a cyclodextrin polymer-functionalized polyaniline/MXene composite was prepared, its electrochemical properties were evaluated for SCs and the authors achieved a Csp of 523.8 F/g. Varghese et al. [132] found that a Csp of 430 F/g can be obtained for MXene/PANI composite-based SCs, which is higher than that of MXene/Ppy (305 F/g) and pristine MXene (105 F/g). Li et al. [133] proposed the fabrication of polylactic acid (PLA)/PANI/MXene (PPM)-based electrodes for the development of flexible SCs, which yielded a Csp of 290.8 F/g at 1 A/g.

### 3.5. MXenes/Carbon-Based Materials for SCs

Wang et al. [134] reported the formation of a novel composite, i.e., polylactic acid (PLA)/polyaniline (PANI)/MXene (PPM), under benign conditions. The authors found a Csp of 290.8 F/g (1 A/g) under optimized conditions. In another study, Olatoye et al. [135] utilized Ni-ZIF-67 (NZ)-based composites with MXene for the development of high-performance SCs. The optimized conditions showed that a Csp of 557 C/g at 0.5 A/g with 5000 cycles can be obtained, whereas An et al. [136] found that a Csp of 346 F/g can be achieved using a CGO/PDAAQ(poly (1,5-diaminoanthraquinone))@MXene composite as the electrode material. The CGO stands for cetyltrimethylammonium bromide (CTAB)-modified graphite oxide. Liu et al. [137] prepared MXene-TiC/rGO sponge composites, which showed the presence of excellent electrical conductivity, an enormous specific surface area with abundant accessible electro-active sites and superior electrochemical properties. The specific capacity of 501 mAh/g was obtained at 0.2 A/g. Li et al. [138] used MXene/CNTs@Ni as electrode modifiers for SCs, and electrochemical investigations suggested that a Csp of 990.8 F/cm^3^ can be obtained under the optimized conditions reported by the authors. Murugan et al. [139] prepared holey carbon nanotube (h-CNT)/MXene hybrid composites for SCs. The h-CNT-wrapped MXene composite showed acceptable capacitance and stability. Moreover, 15 wt% h-CNTs with MXene show the highest Csp of 404 F/g at 4 A/g in a 2 M KOH electrolyte system. Jorn-am et al. [140] reported the fabrication of novel 0D, 1D, 2D and 3D nanostructured materials for SC applications. In this context, the authors prepared activated carbon (AC), and nanowire nickel-doped copper hydroxide compounds (NiDMR) were adopted as the electrode materials, whereas MXenes and CDs were used as the diffusion and surface process enhancers, respectively. This study showed the acceptable electrochemical performance of the proposed SCs. Nasrin et al. [141] explored the use of N-(Nb_2_C/rGO) as an energy storage material and achieved a Csp of 816 F/g, whereas Zhu et al. [142] obtained a Csp of 2.58 mF/cm^2^ using laser-scribed graphene (LSG)/MXene material. In another study, Lyu et al. [143] also adopted Ti_3_C_2_T*_x_*/SWCNT/CNF as electrodes for SCs and achieved a Csp of 746.68 mF/cm^2^ with 10,000 stability cycles. Shi et al. [144] proposed the construction of 3D MXene@rGO, which showed area capacitance of 4.33 F/cm^2^ (10 mA/cm^2^) with 10,000 cycles. Luo et al. [145] proposed the synthesis of MXene/rGO/CNTs (MGC) using a vacuum-assisted filtration method. The MGC-based SCs showed a Csp of 463.5 F/g at 1 A/g and 8000 cycles. Yao et al. [146] proposed the formation of carbonized wood/MXene (CW/MXene)-based electrodes for SC applications. An Csp of 203.94 F/g was obtained at 1 mA/cm^2^. In another study, Nasrin et al. [147] reported the construction of Nb_2_C/BCN (NBCN)-based electrodes for SC applications and observed that a Csp of 765 F/g (2 A/g) can be obtained for NBCN-based SCs. Dharmasiri et al. [148] found a Csp of 908 mF/g at 0.5 mA/g for MXene/CF-based SCs, whereas Yu et al. [149] observed a Csp of 108 F/g at 1 A/g using graphene/MXene-based SCs. Chen et al. [150] also reported a novel MXene-based electrode material (MXene/MWCNT/2,2,6,6-tetramethylpiperidine-1-oxyl (TEMPO) radical-mediated oxidized cellulose nanofibers (TOCNFs) (MMT)) for SC applications.

**Figure 10:**
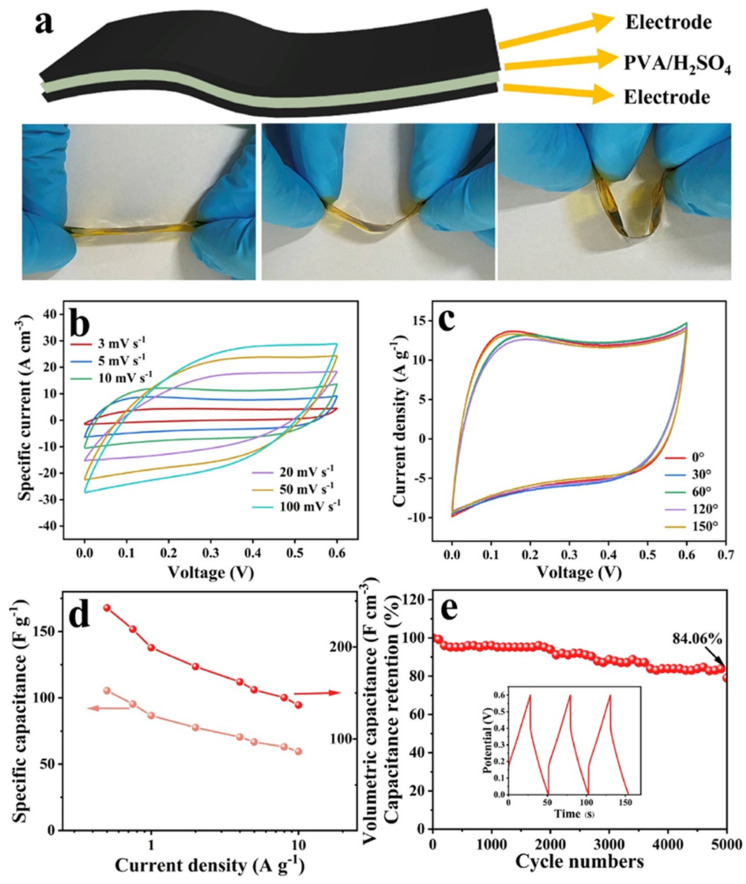
(**a**) Schematic and digital picture of MMT-8 film-based ASSCs. (**b**) CVs of ASSCs at (varied scan rates). (**c**) CVs data of ASSCs at various bending angles. (**d**) Specific capacitance at various adopted current densities. (**e**) Cyclic stability results for ASSCs. Reproduced with permission from reference [150].

The authors developed flexible all-solid-state supercapacitors (ASSCs) using the above-mentioned electrode material. The schematic picture of the flexible ASSCs is displayed in Figure 10a.

The performance of the ASSCs was evaluated by recording CV data, as shown in Figure 10b,c. The Csp versus current density data are shown in Figure 10d. The obtained results showed a Csp of 244.13 F/cm^3^ and 137.45 F/cm^3^ at 0.5 A/g and 10 A/g. The proposed ASSCs also exhibited acceptable stability at 5000 cycles, as shown in Figure 10e. Sun et al. [151] reported redox-organic molecule 2, 6-diaminoanthraquinone (DAAQ)-modified MXene (Ti_3_C_2_T*_x_*)/graphene (DAAQ-M/G) composites using novel strategies. The authors reported a Csp of 226 F/g at 1 A/g for DAAQ-M/G-based SCs with an energy density of 43 Wh/kg at a power density of 1669 W/kg. This work also reported the presence of excellent stability at 9000 cycles. Yang et al. [152] stated that MXene/rGO may be a suitable electrode material for SCs, showing a Csp of 660 F/g at 0.5 A/g with stability at 10,000 cycles. Alharbi et al. [153] used MoSSe@CNT/MXene as an electrode material for SCs and reported a Csp of 585 F/g, whereas Jin et al. [154] used MXene/graphene/CNTs as an electrode material and obtained a Csp of 1862.5 mF/cm^2^ at 1 mA/cm^2^. Rundla et al. [155] also utilized Ti_3_C_2_T_x_ MXene/rGO composites as an electrode material and obtained a Csp of 357 F/g at 1 A/g for the optimized electrode material ((Ti_3_C_2_T_x_)_90_(rGO)_10_). Liu et al. [156] prepared S, N-MXene/rGO flexible films, which showed a Csp of 2414.6 F/cm^3^ at 1 A/g with stability at 5500 cycles.

### 3.6. MXenes/Hydroxides/MOFs/LDH

Liu et al. [157] reported the formation of MXene/Ni(OH)_2_ on nickel foam (NF) for the construction of SCs. The authors found that MXene/Ni(OH)_2_/NF showed an areal capacitance of 0.223 mAh/cm^2^ at 1 mA/cm^2^ and an energy density of 43.68 Wh/kg at a power density of 424.96 W/kg. Zhu et al. [158] found that cobalt hydroxide (Co(OH)_2_) coupled with Ti_3_C_2_T_x_ MXene/NF has a higher Csp of 1400 F/g, which is higher than that of Co(OH)_2_@NF (1106 F/g) and Ti_3_C_2_T_x_@NF (844 F/g). This may be ascribed to the presence of 2D/2D heterostructures of the LDH/MXene hybrid composite. Huang et al. [159] reported the formation of a hierarchical NF@Ti_3_C_2_T_x_@NiCo layered hydroxide (NF@Mxene@NiCo-LDH) composite and its properties for the development of SCs.

**Figure 11:**
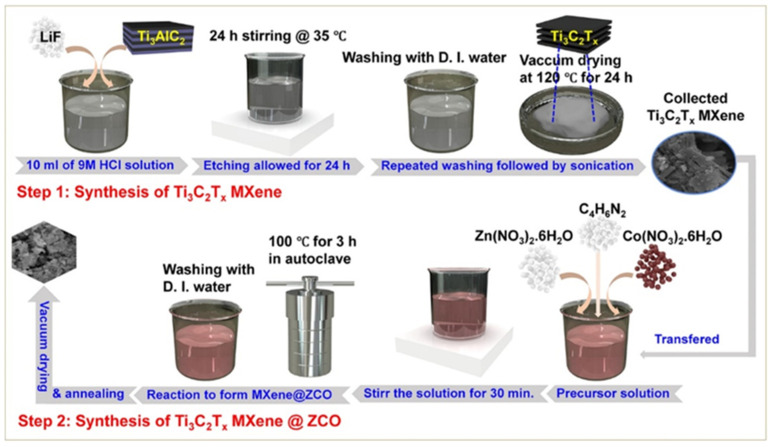
Schematic illustration for the preparation of Ti_3_C_2_T_x_ MXene@ZCO. Reproduced with permission from reference [160].

Kitchamsetti et al. [160] also utilized ZnCo-MOF (ZCM)-derived ZnCo_2_O_4_ (ZCO) particles to adsorb on the (MX) nanosheets and form a mesoporous structure, which may provide flexible ion diffusion pathways. The schematic picture for the preparation of Ti_3_C_2_T_x_ MXene@ZCO is displayed in Figure 11.

**Figure 12:**
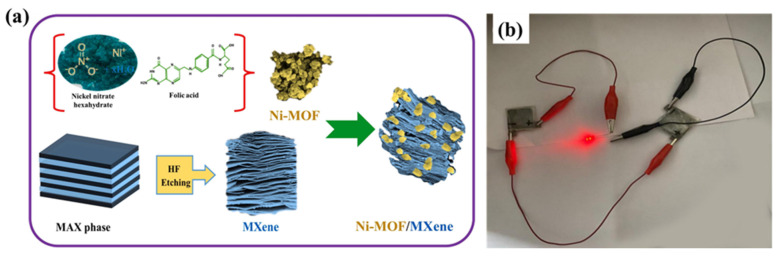
(**a**) Schematic graph shows the synthesis of Ni-MOF/MXene. (**b**) Digital picture shows LED light for Ni-MOF/MXene-based SCs. Reproduced with permission from reference [166].

The synthesized MXene@ZnCo_2_O_4_ (MX@ZCO) composite showed a Csp of 260 mAh/g at 1 mA/g. This work offers an excellent approach for the preparation of MX and ZCO composite material as an electrode modifier for SC applications. Ma et al. [161] proposed the synthesis of NF/MXene/CoAl-LDH, which exhibited a Csp of 646.7 F/g at 0.5 A/g and 450.8 F/g at 5 A/g. Adil et al. [162] adopted FeCu MOF/MXene as an energy storage material, and electrochemical studies showed that a Csp of 440 mAh/g can be obtained under a three-electrode system. Yue et al. [163] found that a Csp of 1924 F/g can be obtained at 0.5 A/g using Ni/Co-MOF@TCT-NH_2_ as an electrode material, whereas Xiang et al. [164] stated that an energy density of 51 Wh/kg at 1.59 kW/kg can be obtained using Zn-Co MOFs@MXene. Ji et al. [165] reported an acceptable Csp of 1160.5 F/g and 736 F/g at 1 and 20 A/g, respectively, using Ni-MOF@MX2 as an electrode material. Shivade et al. [166] proposed the fabrication of Ni-MOF/MXene for its applications in SCs. The synthesis of the Ni-MOF/MXene can be understood from Figure 12a. The authors found that Ni-MOF/MXene-based SCs have a Csp of 716.19 F/g at 1 A/g. Figure 12b shows the digital picture of the LED light connected with SCs.

Another study by Shalini et al. [167] reported a Csp of 1028 C/g at 1 A/g for MXene-MnNi-based SCs. Wang et al. [168] employed the co-precipitation method to prepare the MXene/NiMn LDH composite for SC applications. This work reported a Csp of 1530 F/g at 2 A/g with stability at 20,000 cycles. Lan et al. [169] obtained a Csp of 1210.55 F/g at 1 A/g for the NiCo-LDH@MT-based electrode for the development of SCs. This study also stated that the proposed material has decent stability at 10,000 cycles. Jiang et al. [170] proposed a novel FeCo LDH/MXene composite as a supercapacitor material. The authors obtained a Csp of 2058.2 F/g at 1 A/g and 1732.7 F/g at 10 A/g for the proposed SCs.

### 3.7. Others

**Figure 13:**
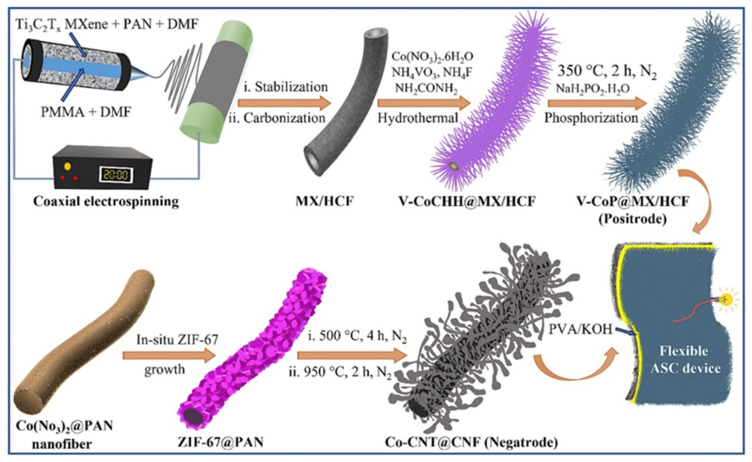
Schematic picture shows the fabrication of MX/HCF, V-CoP@MX/HCF (positrode) and Co-CNT@CNF (negatrode) for ASCs. Reproduced with permission from reference [176].

In other studies, Chen et al. [171] achieved a Csp of 1729 mF/cm^2^ at 0.5 mA/cm^2^ using Ti3C2-based materials, whereas Falak et al. [172] reported the use of MXene/Ag NPs/rGO composite for SCs, which shows a Csp of around 630 F/g with stability at 5000 cycles. Yesilbag et al. [173] optimized the percentage (10%, 20% and 30%) of boron carbon nitride (BCN) and found that MXene/BCN_10_ (m-MX/BCN_10_) is a highly active material for SCs, and a Csp of 678 F/g was observed. Beknalkar et al. [174] used Ag-embedded CoFe-phosphate (CFPAg) and Ti_3_C_2_ MXene as the positive and negative electrodes for hybrid SCs. The proposed work demonstrated a Csp of 1021 mF/cm^2^ with 10,000 stability cycles. Zhang et al. [175] prepared MXene/Co-TCPP composites (CoMX) by using electrostatic self-assembly. The authors incorporated Co-TCPP MXene layers and successfully enhanced ion accessibility, which increases electrochemical performance for SCs. The enhanced Csp of 1591.7 mF/cm^2^ and stability with 8000 cycles were obtained. Pathak et al. [176] demonstrated the preparation of Ti_3_C_2_T_X_ MXene-aligned hollow carbon fiber (MX/HCF)/vanadium-doped cobalt phosphide nanorod arrays (V-CoP@MX/HCF) for asymmetric supercapacitors (ASCs). The formation of MX/HCF, V-CoP@MX/HCF (positrode) and Co-CNT@CNF (negatrode) is demonstrated in Figure 13. The authors found that the proposed ASCs have a Csp of 1896.8 F/g.

Das et al. [177] used an MXene/silica composite as an energy storage material, which exhibited a Csp of 718 Fg^−1^ in 1 M H_2_SO_4_ electrolyte using a three-electrode setup. The authors also developed SCs device with a Csp of 648 Fg^−1^ at 1 Ag^−1^. Chu et al. [178] prepared NW/polyoxometalate (P_2_W_17_Ni)/Ti_3_C_2_T*_x_* for SC applications and achieved a Csp of 16.1 mF/cm^2^ at 0.6 mA/cm^2^. Baasanjav et al. [179] also reported the synthesis of 2D MXene/Ni-Co phosphide (MX/NCP) hybrid composite for SCs using a benign hydrothermal method followed by phosphorization. The synthesized material was used for SCs, which exhibited a Csp of 1754.0 F/g at 3 mA/cm^2^. The electrochemical performance of the MXene-based materials for SCs is summarized in Table 1.

The above results show that MXene with metal oxides and metal sulfides exhibits improved cyclic stability, which may be ascribed to various factors, such as the presence of synergism, active sites, larger surface area and enhanced electrical conductivity of the composite materials. It is also believed that the presence of metal oxides reduces or inhibits the restacking of the MXene nanosheets and provides active sites for redox reactions. Thus, improved ion diffusion and electrochemical performance are observed for MXene-based hybrid composites in SC applications. Similarly, metal sulfides with a layered structure and acceptable electrical conductivity provide a better pathway for redox reactions and improve the ion diffusion and charge storage properties of the MXene-based electrode materials for SCs.

## 4. Electrochemical Sensors

### 4.1. MXenes/Metal Oxides Based Sensors

**Figure 14:**
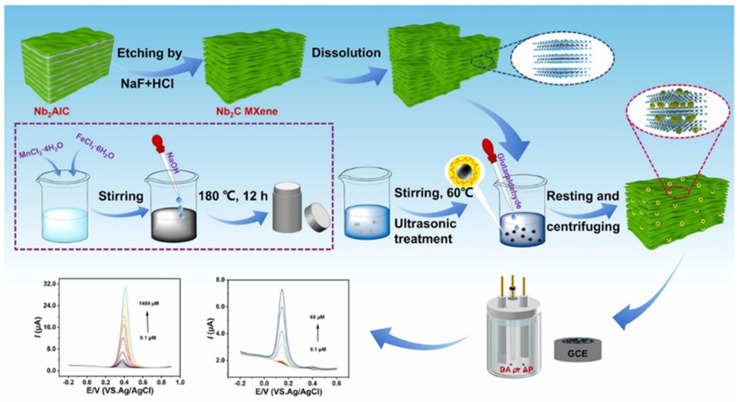
Schematic picture displays the experimental process for the fabrication of AP and DA sensor via three-electrode system. Reproduced with permission from reference [182]. Inset shows preparation of MnFe_2_O_4_.

MXene-based materials are widely adopted for a variety of electrochemical applications, including electrochemical sensors. Previously, Myndru et al. [180] reported the construction of a glucose (Glu) sensor by employing electrochemical sensing technology. The skin-attachable Glu sensor was developed using a ZnO/MXene electrode material. The fabricated sensor showed a limit of detection (LOD) of 17 µM and excellent selectivity in the presence of various interfering compounds via a chronoamperometry (CA) method. The authors also observed that the proposed sensor has good recovery of the real sample in a finger-prick blood sample. In another study [181], the differential pulse voltammetry (DPV) technique was adopted as an electrochemical sensing technology for the detection of bisphenol A (BPA). The authors prepared a V_2_O_5_@Ti_3_C_2_T_x_ composite, explored its catalytic properties for the monitoring of BPA using the DPV method and reported an LOD of 87 nm with a linear range of 411 nm to 31.2 µM. This suggests that the presence of synergistic effects between V_2_O_5_ and Ti_3_C_2_T_x_ enhances the PBA sensing via the DPV method. In another study [182], a Schottky junction-based electrochemical sensor was also developed by using niobium carbide (Nb_2_C) MXene-integrated manganese ferrite (MnFe_2_O_4_) as an electrode material (Figure 14). A MnFe_2_O_4_/Nb_2_C MXene-based electrode was used for the sensing of acetaminophen (AP) and dopamine (DA) via the DPV method. The authors reported LODs of 0.079 and 0.070 µM for the detection of AP and DA, respectively. Alanazi et al. [183] also explored the use of a MXene/rGO composite-modified copper oxide composite for the sensing of Glu via the CA method. The constructed electrode exhibited a low detection limit, along with good selectivity, excellent stability and reproducibility, making it a promising candidate for the monitoring of Glu in human serum samples. These observations suggested that the 3D network of MXene and rGO nanosheets may have enhanced charge transfer activity and boosted the electrochemical sensing performance of the proposed non-enzymatic Glu sensor.

**Figure 15:**
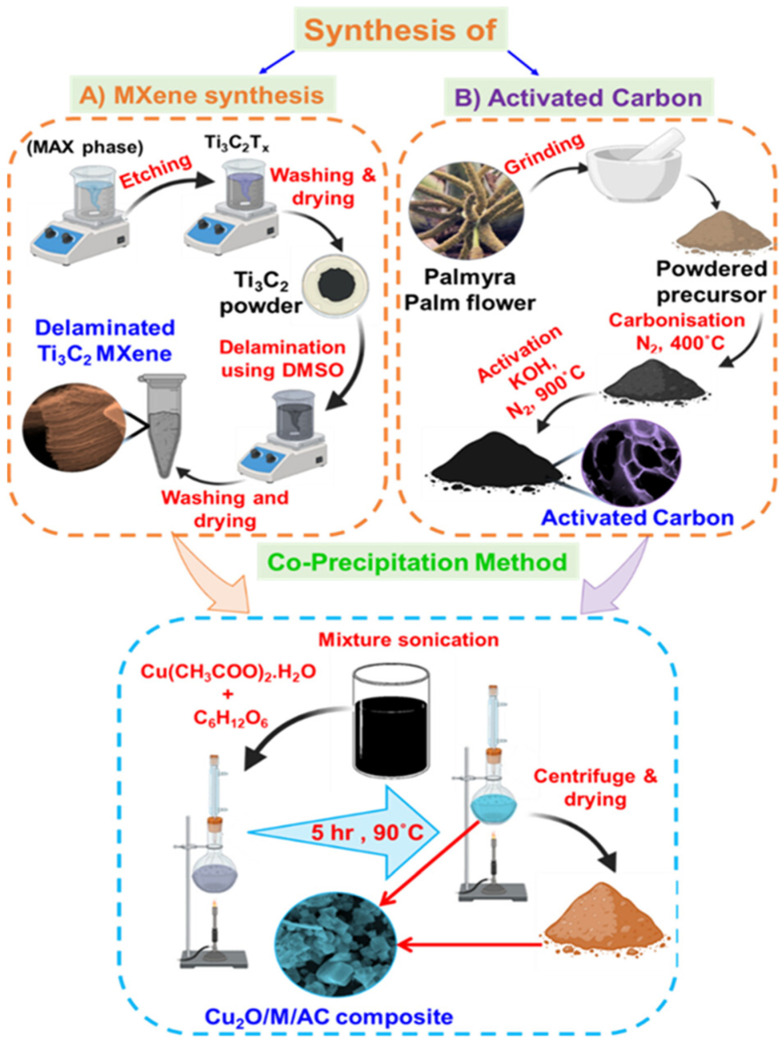
Schematic representation of the formation of Cu_2_O/M/AC composite. Reproduced with permission from reference [184].

Gopal et al. [184] explored a MXene-embedded porous carbon-based Cu_2_O (Cu_2_O/M/AC) composite as a Glu sensing material, and the proposed material was synthesized using the co-precipitation method. Figure 15 shows the schematic representation of the formation of Cu_2_O/M/AC composite. The authors found that the presence of MXene and Cu_2_O on the electrode surface significantly affects the sensing parameters, and enhanced electrochemical performance was observed via CV, EIS and amperometry methods. A decent LOD of 1.96 µM and linear ranges of 0.004 to 13.3 mM and 15.3 to 28.4 nM with sensitivities of 430.3 and 240.5 μA.mM^–1^.cm^–2^ were obtained for the sensing of Glu.

**Figure 16:**
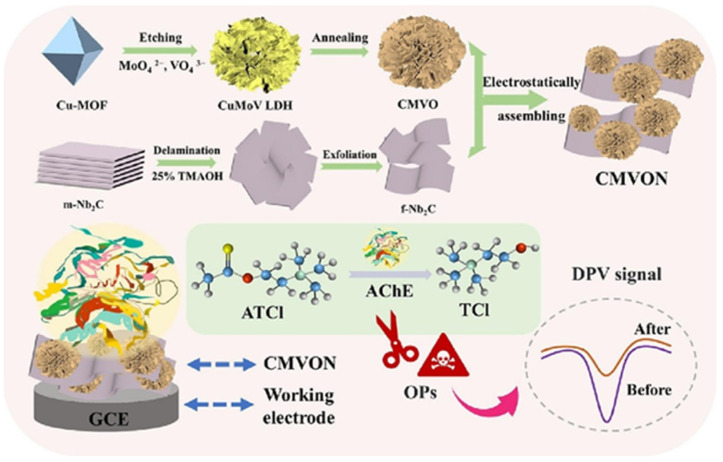
Schematic representation of the fabrication of AChE-CS/CMVON/GCE biosensor for the determination of organophosphorus pesticides (OPs). Reproduced with permission from reference [187].

Prasanna et al. [185] reported that metol (MTL) is widely used in various applications but has negative impacts on aquatic and human life, including the environment. Therefore, the authors proposed the fabrication of an MTL sensor by employing Au/pyrochlore cerium stannate (Au@Ce_2_Sn_2_O_7_)/MXene as an electrode material. This sensor displayed a wide linear range of 0.00125 to 1021.96 µM, an LOD of 5.63 nM and an acceptable sensitivity of 0.403 μA·μM^–1^·cm^–2^. The electrochemical studies also suggested the presence of good selectivity and reproducibility with acceptable stability. It is understood that roxarsone (ROX) is a well-known anticoccidial drug that is excreted in urine and feces, potentially disrupting natural habitats [186]. Baskaran et al. [186] reported the preparation of 2D zinc molybdate-decorated MXene (ZnMoO_4_/MXene) for the determination of ROX using electrochemical methods. It was found that the proposed electrode material (ZnMoO_4_/MXene) exhibits excellent electrocatalytic performance due to its rapid electron transfer rate and higher electrical conductivity. The ZnMoO_4_/MXene/GCE showed high sensitivity (10.413 μA.μM^−1^.cm^−2^) and an appreciable LOD of 0.0081 μM. The electrochemical investigations also suggested the presence of excellent anti-interfering properties for the detection of ROX with reasonably good reproducibility and acceptable recoveries in real samples. Guo et al. [187] proposed the fabrication of a novel hierarchical Cu_3_V_2_O_8_/Cu_6_Mo_5_O_18_ (CMVO) and a few-layer Nb_2_CT_x_ MXene (f-Nb_2_C) composite, and the synthesized sample was labeled as CMVON. The fabrication of the biosensor is presented in Figure 16.

**Figure 17:**
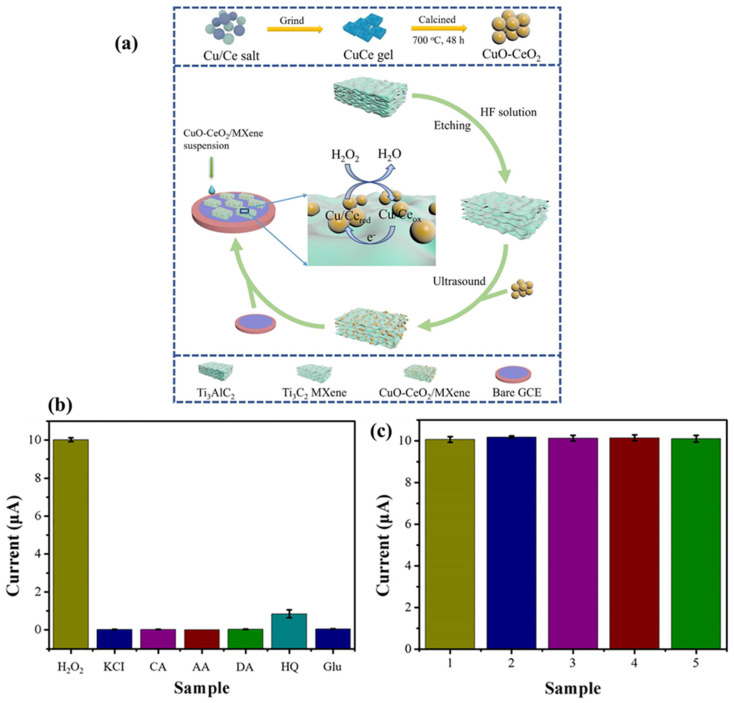
(**a**) Schematic graphs show the preparation of CuO-CeO_2_ and CuO-CeO_2_/MXene. Selectivity (**b**) and reproducibility (**c**) data. Reproduced with permission from reference [191].

The constructed electrochemical sensor (acetylcholinesterase (AChE)-CS/CMVON/GCE) shows an LOD of 2.3 × 10^−14^ with a linear range of 3.6 × 10^−13^ to 3.6 × 10^−8^ M for the detection of fenitrothion, whereas an LOD of 3.0 × 10^−15^ M with a linear range of 3.1 × 10^−14^ to 3.1 × 10^−12^ M was obtained for the sensing of malathion. The proposed sensor also showed an excellent LOD of 3.2 × 10^−16^ M and a linear range of 7.6 × 10^−15^ to 7.6 × 10^−10^ M for the determination of methyl parathion. Quercetin (QRT) is a bioflavonoid with significant biological activities and is beneficial for health [188]. However, the monitoring of QRT is of great significance. In this regard, Ganjeh et al. [188] utilized MXene/copper ferrite (CuFe_2_O_4_) as an electrode material and fabricated a QRT sensor, which shows an LOD of 1.6 nM and two linear ranges of 0.005 to 0.7 µM and 0.7 to 10 µM with acceptable repeatability, stability, reproducibility and selectivity. Sundaresan et al. [189] reported the fabrication of a diquat (DQ) sensor by using samarium stannate (Sm_2_Sn_2_O_7_)-anchored MXene as an electrode modifier. DQ has negative effects on the environment and aquatic life due to its toxicity. This fabricated low-cost DQ sensor displayed acceptable recovery in spinach, bell pepper and tap and river water samples. The proposed DQ sensor also exhibits an LOD of 0.66 nM, a linear range of 0.001 to 2.25 µM, good reproducibility and selectivity, and sensitivity of 0.149 μA·μM^–1^·cm^–2^. Baskaran et al. [190] stated that listeriosis can happen in humans due to listeria monocytogenes. Thus, the authors fabricated electrochemical sensor for the detection of listeria monocytogenes. The polydopamine/zinc molybdate/MXene (PDA@ZnMoO_4_/MXene) exhibited an LOD of 12 CFU/mL with reasonable anti-interfering properties. Zhou et al. [191] reported the fabrication of a hydrogen peroxide (H_2_O_2_) electrochemical sensor by using CuO-cerium oxide (CeO_2_)/MXene as the electrochemical sensing material. The formation of the CuO-CeO_2_ and CuO-CeO_2_/MXene is displayed in Figure 17a. The authors obtained a LOD of 1.67 µM and a good linear range of 5 µM to 100 µM for the sensing of H_2_O_2_. It was also found that the above sensor has excellent selectivity (Figure 17b) and reproducibility (Figure 17c), making it a suitable candidate for practical applications. It is clear that the proposed electrode is highly sensitive and selective to H_2_O_2_ in the presence of various analytes, including citric acid (CA), ascorbic acid (AA), dopamine (DA), Glu and hydroquinone (HQ) (Figure 17b).

### 4.2. MXenes/Metal Sulfides Based Sensors 

**Figure 18:**
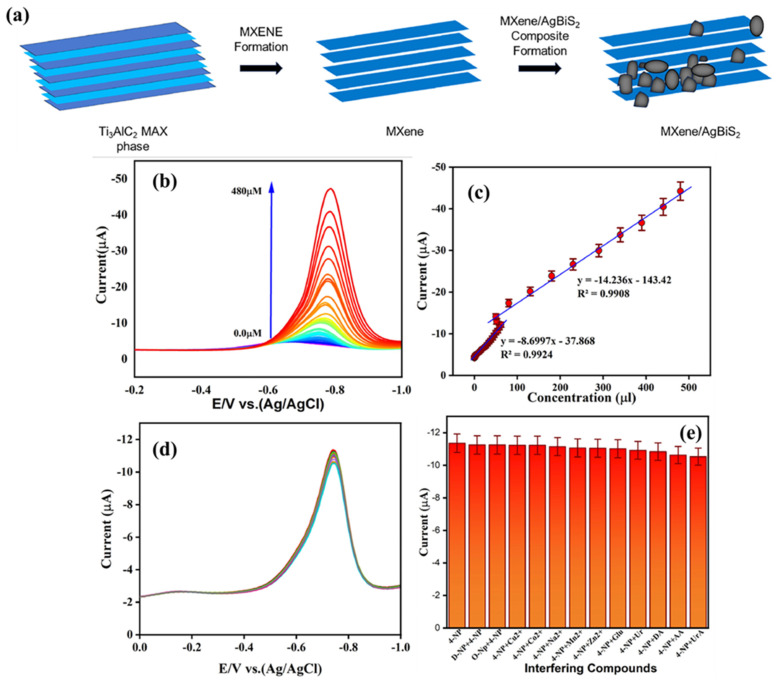
(**a**) Schematic representation of the preparation of MXene/AgBiS_2_ composite. (**b**) DPV curves of the MXene/AgBiS_2_/GCE in presence of various concentrations of 4-NP (0 to 480 nM) at 50 mV/s. (**c**) Calibration plot between current and concentration of 4-NP. (**d**) DPV curves and (**e**) current response for selectivity test. Reproduced with permission from reference [195].

Metal sulfides have decent catalytic properties but suffer from low conductivity, which needs to be addressed before their applications in electrochemical sensing and storage devices. In this regard, Zhang et al. [192] fabricated an ascorbic acid (AA) sensor using a MXene/MoS_2_-modified electrode as an electrochemical sensor and reported a LOD of 4.2 µM and a sensitivity of 54.6 nA μM^−1^. In another study [193], 3D carbon fiber paper (CFP)-MXene-MoS_2_ (CMM) was also utilized as an electrode modifier for the development of AA, dopamine (DA) and uric acid (UA). This sensor exhibited LODs of 1.47 µM, 0.27 µM and 0.38 µM for the determination of AA, DA and UA, respectively. A linear range of 10 to 3000 µM was observed for the sensing of AA, whereas a linear range of 0.5 to 1000 µM was obtained for the sensing of DA and UA. Wang et al. [194] reported the fabrication of AChE entrapped Cs on GCE with platinum/MoS_2_/Ti_3_C_2_ MXene (Pt/MoS_2_/TM) for the sensing of chlorpyrifos. It is worth mentioning that Pt/MoS_2_/TM demonstrates good electrical conductivity, high surface area and good biocompatibility. It was observed that AChE-Cs/Pt/MoS_2_/TM/GCE can be used as an efficient chlorpyrifos sensor, which displayed an LOD of 4.71 × 10^−13^ M and a linear range of 10^−12^ to 10^−6^ M. Recoveries of 94.81% to 104% were observed for real sample studies of fruits and vegetables samples. 4-Nitrophenol (4-NP) poses significant toxic and hazardous effects to the environment and aquatic life, necessitating the development of a 4-NP sensor using reliable analytical techniques. Gopi et al. [195] prepared a MXene/silver bismuth sulfide (AgBiS_2_) composite and deposited it on the surface of GCE for the construction of a 4-NP sensor. The preparation of the MXene/AgBiS_2_ composite is illustrated in Figure 18a. The synthesized material was deposited on the GCE surface for the sensing of 4-NP. The authors found that the current increases with an increase in the concentration of 4-NP (Figure 18b). This current response linearly increases as suggested by the calibration curve between the current and the concentration of 4-NP (Figure 18c). This 4-NP sensor exhibits improved electrochemical performance in terms of the LOD (0.00254 µM), sensitivity (5.862 µA µM^−1^ cm^−2^), selectivity and linear range (0.02 to 1869 µM). The authors found that the proposed sensor has excellent anti-interfering properties for the determination of 4-NP in the presence of other analytes, including UA, AA, DA, Glu, Mn, Co^2+^, Cu^2+^, etc. (Figure 18d,e). The real sample investigations also suggested its potential for large-scale applications.

Mashhadizadeh et al. [196] proposed the SPCE/*N*-MPG/CuS flower-like/MXene-based nalbuphine (NAL) sensor. This sensor showed an LOD of 1.6 µM with a linear range of 5 to 150 µM for the determination of NAL using the DPV method. Zhang et al. [197] reported the construction of a phoxim sensor using simple strategies. The authors prepared Ti_3_C_2_/MoS_2_@AuNPs/AChE for the sensing of phoxim and achieved an LOD of 5.29 × 10^−15^ M and a linear range of 1 × 10^−13^ M to 1 × 10^−7^ M. It was observed that the presence of synergistic interactions was responsible for the improved sensing performance.

### 4.3. MXenes/Polymers Based Sensors

Neampet et al. [198] combined Pt NPs, polyaniline (PANI) and Ti_3_C_2_ MXene and deposited them on a screen-printed carbon electrode (SPCE) for the sensing of H_2_O_2_ and lactate. This sensor showed a LOD of 1 µM for H_2_O_2_, whereas it was 5 µM for lactate. This sensor was also efficient for the sensing of lactate in milk samples, showing stability and reliability. Cheng et al. [199] synthesized PANI-Ti_3_C_2_ composite for the detection of mercury (Hg^2+^) using an electrochemical method. This prepared sensor demonstrated an LOD of 0.017 µg/L with a linear range of 0.1 to 20 µg/L for the monitoring of Hg^2+^. The authors also stated that the Hg^2+^ sensor is a promising electrochemical sensor for real sample studies on tap water. Lu et al. [200] adopted a molecularly imprinted polymer (MIP) approach for the construction of a catechol (CC) sensor. In this regard, amino-functionalization bimetallic organic framework materials (Fe@Ti-MOF-NH_2_) were integrated with MXene and combined with polythionine (pTHi) and MIP. The MIP/pTHi/MXene/Fe@Ti-MOF-NH_2_/GCE shows a LOD of 0.54 µM and linear ranges of 1 to 300 µM and 300 to 4000 µM. Owing to the synergistic interactions in the fabricated electrode material, the developed sensor also exhibited excellent reproducibility and selectivity. L-tryptophan (L-Trp) is crucial for human metabolism, and its imbalance or deficiency may cause various diseases, such as insomnia, depression and heart disease. The monitoring of L-Trp is of great significance, and Zhang et al. [201] developed an electrochemical sensor for the detection of L-Trp. The polyoxometalate (P_2_Mo_17_V)/Ti_3_C_2_T_x_ MXene/Cs was prepared by employing a layer-by-layer self-assembly strategy to construct the electrochemical sensor for the determination of L-Trp. The authors observed that the prepared composite film shows improved electron transfer and excellent electrocatalytic properties for the sensing of L-Trp with a linear range of 0.1 to 103 μM, reproducibility, selectivity, an LOD of 0.08 μM and good repeatability. The authors also reported excellent sensing performance in milk samples with a recovery of 95.78 to 104.31%. Saraswathi et al. [202] focus on the preparation of a NiO NPs/PANI/Ti_3_C_2_T*_x_* (NiOMP) composite for the construction of a Glu sensor. The authors employed CV and DPV techniques for the detection of Glu at the surface of the NiOMP-based electrode. The NiOMP-based Glu sensor exhibited an LOD of 0.019 µM and a sensitivity of 3551.53 µA mM^−1^ cm^−2^ with a linear range of 5 to 500 µM.

**Figure 19:**
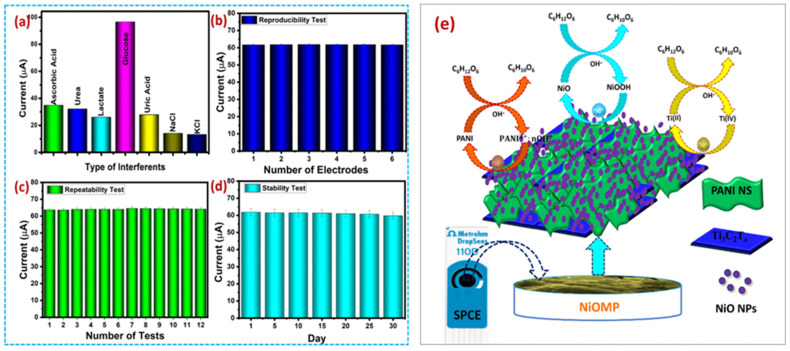
(**a**) Selectivity, (**b**) repeatability, (**c**) reproducibility and (**d**) stability of NiOMP/SPCE for the determination of Glu. (**e**) Probable sensing mechanism for the detection of Glu. Reproduced with permission from reference [202].

The proposed Glu sensor also showed excellent selectivity (Figure 19a), reproducibility (Figure 19b), repeatability (Figure 19c) and stability at 30 days (Figure 19d). The probable working mechanism for the determination of Glu is described in Figure 19e. In another study, Khaleque et al. [203] developed a ciprofloxacin sensor by utilizing poly (rutin)/Ti_3_C_2_T_x_ as an electrode modifier. The authors reported a decent LOD of 1 nM and a sensitivity of 0.49 μA/μMcm^2^ with a linear range of 1.0 × 10^−9^–1.0 × 10^−4^ molL^−1^ for the monitoring of ciprofloxacin. This ciprofloxacin sensor was also efficient for real sample studies in blood serum, suggesting its potential for practical applications.

### 4.4. MXenes/Carbon-Based Materials for Sensors 

**Figure 20:**
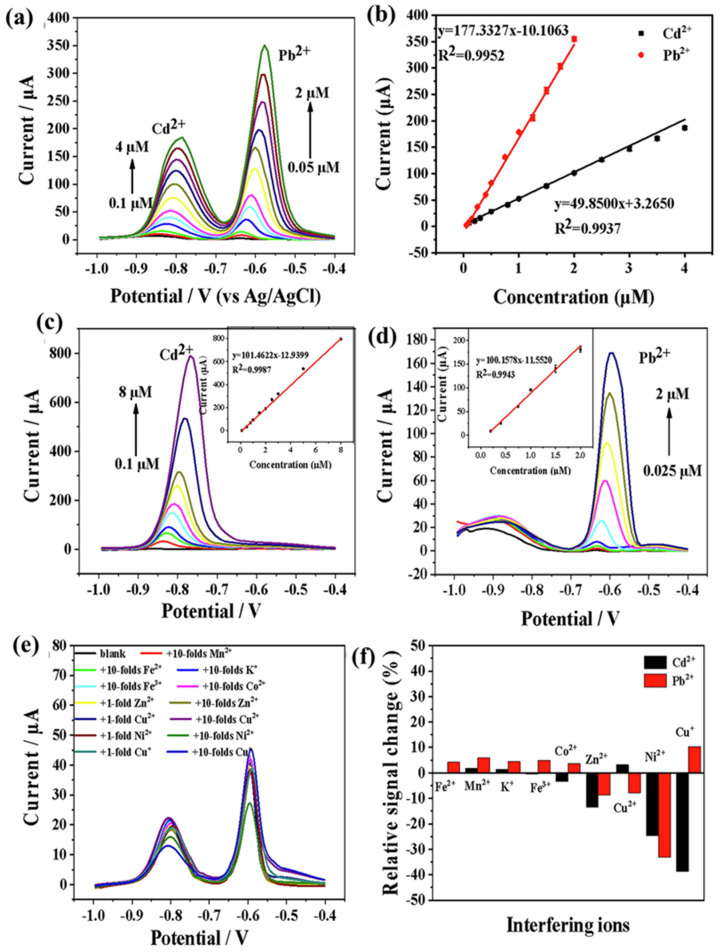
(**a**) SWASV graphs of Ti_3_C_2_@N-C/GCE in presence of Pb^2+^ (0.05 to 2 µM) and Cd^2+^ (0.1 to 4 µM). (**b**) Calibration curve between current and concentration of analyte. SWASV curves of Ti_3_C_2_@N-C/GCE in presence of (**c**) Cd^2+^ (0.1 to 8 µM) and (**d**) Pb^2+^ (0.025 to 2 µM). Inset shows corresponding calibration curve. (**e**) SWASV curves for selectivity test and (**f**) effect of interfering substances on current response. Reproduced with permission from reference [207].

Huang et al. [204] reported the fabrication of a CC and hydroquinone (HQ) sensor by developing a novel electrode. The electrode material consisted of Ti_3_C_2_ and multi-walled carbon nanotubes (MWCNTs). The fabricated electrode showed LODs of 6.6 nM and 3.9 nM for CC and HQ, respectively. However, the linear range (2 to 150 µM) was found to be the same for CC and HQ. Additionally, this electrode was found to be stable, reproducible and selective for the determination of CC and HQ using DPV methods. The real industrial wastewater was also used as a real sample, and the proposed electrode showed excellent recoveries of CC and HQ at 96.9 to 104.7% and 93.1 to 109.9%, respectively. In another study [205], a Ti_3_C_2_T_x_ Mxene/LBG (laser-burned graphene)-based sensor was also reported for the sensing of cortisol, which showed an LOD of 1 pg/mL and a linear range of 0.01 to 100 nM. Chen et al. [206] utilized a polydopamine-functionalized MXene (MXene@PDA/NH_2_-MWCNTs) composite as an electrode modifier and constructed an AP sensor. This AP sensor demonstrated an excellent LOD of 1 nM and two linear ranges of 5 nM to 10 µM and 10 µM to 60 µM, with reasonably good selectivity, reproducibility and stability. The synergistic interactions between the MXene@PDA and NH_2_-MWCNTs may improve the sensing performance of the proposed AP sensor. As per the previous report by Zhang et al. [207], it was observed that heavy metal ions (HMIs) can be efficiently detected by employing N-doped carbon-coated Ti_3_C_2_ MXene as an electrode material. The synthesized material has been labeled as Ti_3_C_2_@N-C. This material was deposited on the GCE surface and explored for the sensing of cadmium (Cd^2+^) and lead (Pb^2+^). The authors adopted square wave anodic stripping voltammetry (SWASV) for the determination of Cd^2+^ and Pb^2+^ in tap and sea water samples. The SWASV curves of the Ti_3_C_2_@N-C/GCE in the presence of Pb^2+^ (0.05 to 2 µM) and Cd^2+^ (0.1 to 4 µM) are displayed in Figure 20a. It can be seen that the current response linearly increases with an increasing concentration of Cd^2+^ and Pb^2+^ (Figure 20b). This suggests that Ti_3_C_2_@N-C/GCE can be used as an electrochemical sensor for the simultaneous detection of Cd^2+^ and Pb^2+^. The SWASV of the Ti_3_C_2_@N-C/GCE was also obtained for various concentrations of Cd^2+^ (0.1 to 8 µM), as shown in Figure 20c. The current response linearly increases with increasing concentration of Cd^2+^ (inset of Figure 20c). Similarly, the concentration of Pb^2+^ was varied in the range of 0.025 to 2 µM, and SWASV curves were obtained (Figure 20d). It was seen that the current response linearly increased with an increasing concentration of Pb^2+^ (inset of Figure 20d). The proposed sensor exhibited LODs of 2.55 nM and 1.10 nM for Cd^2+^ and Pb^2+^, respectively. This sensor was also selective for the sensing of Cd^2+^ and Pb^2+^ in the presence of other metal ions, including Fe^2+^, Cu^+^, Zn^2+^, Co^2+^, K^+^, Fe^3+^, Cu^2+^ and Ni^2+^. It was observed that the presence of the N-C and Ti_3_C_2_ heterostructure enhanced the conductivity and facilitated electron transfer, which further improved the sensing performance of the developed sensor. This sensor also exhibited excellent selectivity for the monitoring of Cd^2+^ and Pb^2+^ (Figure 20e,f).

Ni et al. [208] reported a decent LOD of 3.2 nM and a sensitivity of 16 A/M with a linear range of 0.1 to 30 µM using a Ti_3_C_2_ MXene/graphitized MWCNTs/ZnO-based electrode with excellent selectivity and satisfactory real sample results in human serum samples. This suggests that the proposed sensor can be used in biomedical and clinical applications. Chen et al. [209] also adopted an SPCE as a substrate, and its surface was modified with 3D melamine-doped GO/MXene aerogel (3D MGMA). The 3D MGMA-modified SPCE demonstrated excellent selectivity for the sensing of Cd^2+^, Zn^2+^ and Pb^2+^ with LODs of 0.45 μg L^−1^, 0.48 μg L^−1^ and 0.29 μg L^−1^, respectively. The authors also used tap water, river water and lake water for real sample studies, and experimental investigations showed satisfactory results. Qu et al. [210] reported the fabrication of a novel electrode for the sensing of BPA and dimethyl bisphenol A (DM-BPA). The SPCE was modified with Pt@SWCNTs/Ti_3_C_2_/rGO, and its electrochemical sensing properties were examined for the determination of BPA and DM-BPA. LODs of 2.8 and 3 nM were observed for the monitoring of BPA and DM-BPA, respectively, with excellent selectivity and stability. Dong et al. [211] developed an electrochemical sensor using MXene@rGO aerogel-doped UiO-66-NH_2_ as an electrode material for the determination of Cd^2+^ and Pb^2+^. The authors observed that MXene@rGO acted as a structural support for UiO-66-NH_2_ and enhanced the conductivity of the composite by facilitating electron transport in the matrix. It was also stated that UiO-66-NH_2_ easily provides binding sites for HMIs, and the presence of PhNH_2_/PhNH_2_^+^ in the composite may promote the detection of oxidation/reduction processes of the HMIs on the surface of the electrode. The authors obtained LODs of 0.46 and 0.40 ppb for the determination of Cd^2+^ and Pb^2+^, respectively. Ankitha et al. [212] used a flexible conductive carbon yarn (CCY)-modified Ti_3_C_2_T_x_ MXene sensing electrode for the determination of DA, and the obtained results showed excellent reproducibility, an LOD of 316 pM and excellent recovery in a real sample. In another report, Hou et al. [213] prepared N, S-doped carbon dots (N,S-CDs)/Ti_3_C_2_T_x_ composites for the determination of DA using electrochemical methods. This sensor demonstrated a linear range of 1 to 1000 µM and a LOD of 0.91 µM. Wang et al. [214] reported a novel approach for the formation of N, S-MXene quantum dots (N, S-MQD)/rGO and its composite with a defective bimetallic organic framework (D-FeCu-MOF). The synthesized material (D-FeCu-MOF/N,S-MQD@rGO) was used as a CC sensing material, which demonstrated an LOD of 0.0014 µM and a wide linear range of 0.004 to 520 µM. Facure et al. [215] found that Ti_3_C_2_ MXene/GQDs may be a suitable electrode material for the fabrication of a DA sensor. Thus, the authors fabricated a DA sensor and achieved an acceptable LOD of 1.8 µM with a linear range of 40 to 400 µM and excellent recovery in real samples, which suggested its potential for clinical and biomedical applications. Mohammadi et al. [216] also reported the construction of Cu^2+^ and Hg^2+^ sensors using novel strategies. The layered N-doped carbon/MXene was used as a sensing material, and the authors achieved decent LODs of 0.019 and 0.056 µM for the determination of Cu^2+^ and Hg^2+^, respectively. The fabricated sensor also showed sensitivities of 114.54 and 64.317 µA µM^−1^ cm^−2^ for the monitoring of Cu^2+^ and Hg^2+^, respectively. Zhang et al. [217] prepared a novel material by combining few-layer Ti_3_C_2_T_x_ and a zeolitic imidazolate framework-67 to form the Ti_3_C_2_T_x_-ZIF-67 composite. This was further converted to Ti_3_C_2_T_x_-Co@NC using benign strategies. The Ti_3_C_2_T_x_-Co@NC demonstrated an LOD of 66.8 nM and a linear range of 0.5 to 100 µM for the determination of Glu. This excellent sensing performance may be attributed to the presence of synergism, which provided a better electron transfer process and active sites for enhanced electrochemical reactions.

### 4.5. MXenes/MOFs/LDH Based Sensors 

**Figure 21:**
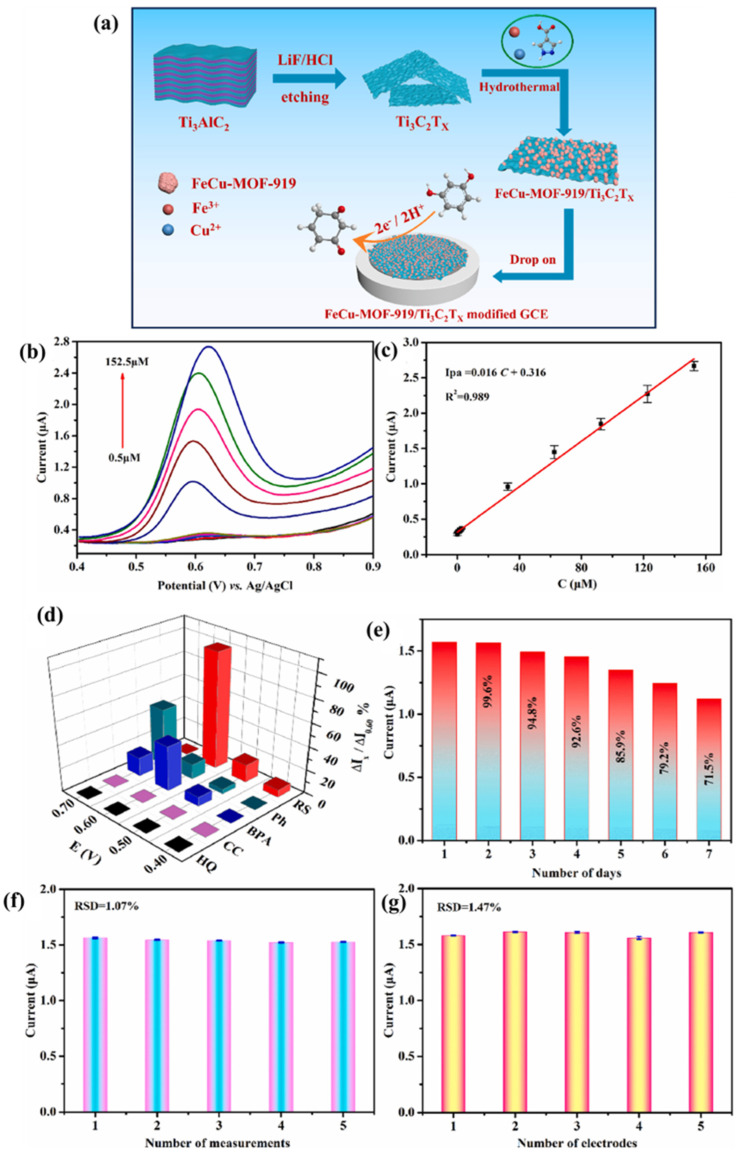
(**a**) Schematic illustration of the preparation of FeCu-MOF919/Ti_3_C_2_T_x_/GCE. (**b**) DPV curves of the FeCu-MOF919/Ti_3_C_2_T_x_/GCE in various concentrations of RS and (**c**) calibration plot between current and concentration of RS. (**d**) Selectivity, (**e**) stability, (**f**) repeatability and (**g**) reproducibility studies. Reproduced with permission from reference [221].

Chen et al. [218] prepared a Ce-MOF/Ti_3_C_2_T_X_ MXene sensing material by employing novel approaches and used it for the determination of L-Trp. It was observed that the combination of Ce-MOF with MXene efficiently hinders the stacking of MXene nanosheets. The proposed L-Trp sensor showed an LOD of 0.19 µM, interfering resistance, reproducibility, with a linear range of 0.2 to 139 µM, real sample recovery in blood serum, and excellent long-term stability. Xiao et al. [219] explored the sensing properties of the Fe-MOF/MXene for the detection of arsenic (As (III)) by using the SWASV method. This sensor displayed sensitivity of 8.94 µA·(ng·L^−1^)^−1^·cm^−2^ with an LOD of 0.58 ng/L. Paul et al. [220] proposed the construction of a DA sensor in the presence of AA and 5-aminovaleric acid (VA). The DA sensor was constructed using an MOF/MXene composite as the electrode material. This sensor displayed LOD of 110 nM and linear range of 90 to 300 nM for the monitoring of DA. Zhang et al. [221] explored a novel FeCu-MOF-919/MXene composite for the sensing of resorcinol (RS). The fabrication of the RS sensor is shown in Figure 21a.

Authors utilized the DPV method for the sensing of RS using FeCu-MOF919/Ti_3_C_2_T_x_/GCE (Figure 21b). The authors found that the current response linearly increases with an increasing concentration of RS (Figure 21c). The FeCu-MOF919/Ti_3_C_2_T_x_/GCE exhibited an LOD of 0.08 µM for the sensing of RS. This sensor also displayed a decent linear range of 0.5 to 152.5 µM and a sensitivity of 0.23 μA·μM^–1^·cm^–2^, with excellent selectivity (Figure 21d), stability (Figure 21e), repeatability (Figure 21f) and reproducibility (Figure 21g). The proposed FeCu-MOF919/Ti_3_C_2_T_x_/GCE was also used for real sample investigations on tap water. The authors found satisfactory recovery for the detection of RS in tap water. This suggests that the presence of the larger surface area of FeCu-MOF919 and the highly conductive nature of Ti_3_C_2_T_x_ enhanced the electrochemical properties of FeCu-MOF919/Ti_3_C_2_T_x_/GCE. Qi et al. [222] developed an ofloxacin (OFL) sensor using Fan e-MOF-NH_2_/CNTs-NH_2_/MXene composite. This OFL sensor displayed a linear relationship of 0.1 μM to 100 μM and an LOD of 13.2 nM. Boruah et al. [223] obtained a decent LOD of 0.2 nM and a linearity of 1 to 100 nM for the determination of DA using an MOF/Nb_4_C_3_T*_x_* MXene-based electrode. Shi et al. [224] proposed the formation of MXene-NH_2_@CeFe-MOF-NH_2_ for the sensing of Cd^2+^, Pb^2+^ and Hg^2+^ and reported decent LODs of 0.69 nM, 0.95 nM and 0.33 nM, respectively. This sensor was also efficient in real samples such as corn, milk, rice and fish samples. Xu et al. [225] reported the fabrication of CoNi ZIF-MXene@MWCNT/CC for the sensing of salidroside (SAL). This sensor exhibited a LOD of 0.0958 µg/mL and a linear range of 0.5 to 500 µg/mL for the sensing of SAL. Hou et al. [226] reported the synthesis of a MXene@PDA/MOF composite with a honeycomb-like morphology for the sensing of l-cysteine (L-Cys) via the DPV technique. This fabricated L-Cys sensor showed an LOD of 3.74 nM and a linear range of 0.01 to 5 µM. The real sample studies also showed acceptable performance for practical applications. Nitrofurantoin (NFT) sensor was developed by Liu et al. [227] using a Ru/NiFe-LDH-MXene-modified SPCE. This developed sensor displayed an excellent sensitivity of 152.44 μA·μM^–1^·cm^–2^ and an LOD of 2.2 nM with satisfactory real sample studies. Shahparast et al. [228] adopted CV, DPV, chronocoulometry (CC) and CA techniques to characterize the electrochemical properties of the FeCu-LDH@MXene. The FeCu-LDH@MXene-based electrode showed an LOD of 90 nM, a sensitivity of 0.0327 µA/µM and a linear range of 0.66 to 418 µM for the monitoring of clonazepam (CLZP). This CLZP sensor also showed satisfactory results in human plasma and pharmaceutical tablet samples.

### 4.6. MXenes/Metal Nanoparticles/Others

Liu et al. [229] prepared PtNP@MXene-Ti_3_C_2_T_x_ for the construction of an L-glutamate sensor. This sensor displayed an LOD of 0.45 µM and a linear range of 10 to 110 µM. Rasheed et al. [230] explored the preparation of Pt@Ti_3_C_2_T_x_ for electrochemical sensing applications. The 10%Pt@Ti_3_C_2_T_x_ displayed better electrochemical properties and an LOD of 32 nM, and a linear range of 50 nM to 5 µM was obtained for the determination of BPA. This sensor was also effective for the monitoring of BPA in fresh milk and drinking water samples. Chandran et al. [231] developed a biosensor for the detection of CC using laccase (Lac)-immobilized Au/Mxene as the electrode material. Lac/Au/Mxene was coated on GCE and explored for the sensing of CC, displaying an LOD of 0.05 µM, a linear range of 0.05 to 0.15 µM and a sensitivity of 0.05 mA/µM. This sensor also has promising features, such as repeatability, reproducibility and selectivity, for the sensing of CC. Kumar et al. [232] developed a methylmalonic acid (MMA) biosensor using a Ni-embedded Ti_3_C_2_T_x_ (MX−Ni)-modified electrode. This MMA sensor displayed an LOD of 0.12 pM and wide linear ranges of 0.001 to 0.003 µM and 0.0035 to 0.017 µM. The authors also found that the proposed sensor is a promising candidate for the determination of MMA in urine samples. Sun et al. [233] reported the fabrication of Au@MXene for the sensing of aflatoxin B1 (AFB1), and the proposed sensor displayed an LOD of 2.8 nM. In other work, Khoshfetrat et al. [234] reported the formation of methyl orange (MO)-delaminated Ti_3_C_2_ MXene using the benign one-step method for the construction of the H_2_O_2_ sensor. The authors found that MO incorporation into MXene facilitates electron transportation and improves the conductivity and surface area. Linear ranges of 0.33 to 1200 µM and 0.1 to 1350 µM, with LODs of 0.05 µM and 0.01 µM, were observed for the sensing of H_2_O_2_ and hydrazine, respectively. This sensor also showed good selectivity. The electrochemical performance of the MXene-based sensors is summarized in Table 2.

The above results show that MXene-based electrochemical sensors are promising electrode materials for the detection of a targeted analyte, with a decent selectivity, LOD and sensitivity. However, the LOD varies and depends on the type of targeted analyte, which is based on the differences in the electrochemical interactions and material properties. It is believed that surface functionalization, heteroatom doping and tailoring the physicochemical properties of MXene by combining it with novel nanomaterials may further improve the sensing properties of MXene-based hybrid materials. Additionally, the formation of heterojunctions should be further studied, and in-depth investigations should be carried out to examine the presence of synergism and electrode kinetics for sensing studies.

## 5. Conclusions, Challenges and Future Perspectives

This review article highlights the potential of MXene and its composites as an advanced electrode modifier for SCs and electrochemical sensing applications. It is worth mentioning that MXenes are highly conductive layered materials and show excellent electrochemical properties, which make them a desirable electrode material for SCs and electrochemical sensing technology. Although MXene and its composites demonstrated excellent capacitance, stability and power density for SCs, and excellent sensitivity, detection limits and selectivity for electrochemical sensors, MXene still has some limitations and drawbacks that need to be considered before they are used in practical or large-scale applications. The synthesis of MXene involves the use of highly corrosive HF, which is itself a hazardous condition to handle during etching procedures. Future research should focus on the design of novel eco-friendly synthetic procedures for the preparation of MXenes. MXene shows excellent conductive properties and electrochemical activities for SCs and sensing applications. However, several challenges exist, which require further research for wide-scale applications.

i. MXene suffers from the restacking of the nanosheets, which is due to the van der Waals forces, which can significantly affect the surface area and ion movements. Thus, the electrochemical performance of the MXene-based electrode materials can be influenced for SCs and sensing studies.
ii. MXene also faces structural degradation and compromised electrochemical performance for long-term stability and cycles for SCs and sensing applications.
iii. The synthesis of MXene involves harsh conditions, such as the use of HF, increasing the cost of the preparation of MXene and resulting in negative impacts on the environment. Thus, etching-based synthetic methods for the preparation of MXene restrict their potential applications to large-scale production.
iv. The surface chemistry for the modification and functionalization of MXene is still unknown and needs in-depth investigations.
v. Scalability is also another challenge for large-scale production.
vi. The toxicity of the fluoride groups on the MXene surface is another challenge.
vii. The preparation of MXene with uniform morphological characteristics and controlled surface properties remains a challenge.
viii. MXene can be oxidized in alkaline electrolyte solution-based systems for long-term operations.

The challenges mentioned above motivated the researchers to combine MXene with metal oxides and polymeric materials to form hybrid composites for SCs and sensing applications. The MXene-based composites displayed improved structural stability and improved electrochemical performance for SCs and sensing applications. This may be ascribed to the presence of synergistic interactions and the decent electrochemical properties of the MXene-based hybrid composites. However, the long-term stability of the MXene-based SCs and sensors remains a challenge. MXene-based hybrid composite materials also have some limitations, including interfacial incompatibility, reduced charge transport and difficulties in achieving uniform dispersion within the composite matrix. These challenges exploit the synergistic effects of the MXene-based hybrid composites. Thus, future research may focus on the challenges discussed above to improve scalability and reduce the cost of the MXene-based materials for electrochemical applications. We also believe that the following key points may be useful for future research directions:

(a) Novel fluoride etching-free and green synthesis methods should be developed for the preparation of MXene instead of conventional methods with harsh conditions (HF etching).
(b) The surface passivation strategy may be applied to improve the structural stability of MXene by incorporating carbon-based or protective polymer-based layers.
(c) In-depth studies are required to understand the mechanism of the formation of MXene-based composites and their charge transfer properties for electrochemical applications.
(d) The synergistic interactions need to be studied in-depth.
(e) The introduction of ionic liquid-based electrolytes or novel electrolyte additives may be useful to improve the stability of MXene-based materials for long-term cycles.
(f) MXene-based materials can be employed for the construction of flexible SCs and sensors due to their excellent mechanochemical properties.

## Figures and Tables

**Table 1:** Electrochemical performance of the MXene-based SCs.

Electrode Modifier	Electrolyte	Retention@Stability Cycles	C_sp_ (F/g)	Current Density (A/g)	Refs.
MXene/NiPc	3 M KOH	95.1%@5000	792	1	[44]
Ti_3_C_2_T_x_	3 M H_2_SO_4_	92%@10,000	426	1	[45]
N-Ti_3_C_2_T_x_	2 M H_2_SO_4_	80.4%@8000	449	1	[46]
Nb_2_C-PCarbons CPs	6 M KOH	93.93%@10,000	465.6	0.5	[47]
Ti_2.9_Nb_0.1_C_2_T_x_	1 M H_2_SO_4_	92.89%@10,000	1014 F/cm	2 mV/s	[48]
1-Co@Ti_3_C_2_T_x_ MXene	1 M KOH	5000	48 mAh/g	1	[51]
Biomass/MXene/Cs aerogel	3 M H_2_SO_4_	82%@50,000	1526.4 mF·cm^3^	2 mA/cm^3^	[52]
SSA@Ti_3_C_2_T_x_	3 M H_2_SO_4_	90.1%@20,000	321	1	[55]
Mg-10%MFMX@MS	3 M H_2_SO_4_	10,000	685.77 mF/cm^2^	10 mV/s	[58]
Mo_2_N MXene	2 M KOH	93.9%@5000	1272.45	10 mV/s	[59]
CoMoO_4_-Ti_3_C_2_T_x_	6 M KOH	68.2%@6000	870.7 C/g	1	[63]
MNF/Ti_3_C_2_T_x_	1 M Na_2_SO_4_	91.9%@5000	348.5	0.5	[66]
MXene-WO_3_@rGO_sp_	2 M KOH + 0.1 M K_4_[Fe(CN)_6_]	86%@3000	774.4	5	[68]
d-Ti_3_CN@NiCeO_2_	2 M KOH	79%@8000	941	1	[69]
CuMn_2_O_4_/Ti_3_C_2_	2 M KOH	80%@10,000	628 mF/cm^2^	4 mA/cm^2^	[70]
MnFe_2_O_4_/MXene	2 M KOH	97.8%@5000	1263	1	[72]
MXene/TiO_2_-G	EmimTFSI-ACN-LiTFSI	85.1%@5000	196.2	1	[75]
d- V_4_C_3_T_x_MoO_3_	3 M H_2_SO_4_	97%@10,000	6450 C/g	1	[77]
40 wt% g-C_3_N_4_/MoO_3_	1 M H_2_SO_4_	96.8%@5000	1168	1	[80]
(CeO_2_/MXene)/PANI(40%:60%)	2 M KOH	96.3%@6000	2247.962	2	[82]
MXene/NiCo_2_S_4_	3 M KOH	96.51%@10,000	2675	1	[84]
MXene/CoFe_2_O_4_/g-C_3_N_4_	3 M KOH	89%@5000	1506.2	5	[86]
NiCo_2_O_4_@MXene	3 M KOH	89.4%@10,000	777.7	1	[88]
WS_2_@MXene/GO	1 M KOH	5000	1111	2	[91]
Ti_3_C_2_T_x_/NH_2_-RGO	1 M KOH	97%@10,000	120.2	1	[95]
CoS/MXene/PANI	1 M H_2_SO_4_	97%@10,000	246	2	[97]
MXene/CuS	3 M KOH	93.5%@10,000	2569.3	1	[99]
CNS/C/MXene	6 M KOH	71.17%@30,000	1221.6	1	[105]
MXene/FeNi_2_S_4_	6 M KOH	90%@2000	673	1	[108]
Ti_3_C_2_T_x_ (MXene)/WS_2_	1 M H_2_SO_4_	-	373	0.4	[111]
MXene/PANI	1 M KOH	95.5%@1000	458.3	5 mV/s	[116]
P-M10	H_2_SO_4_	98.5%@10,000	1196.5	1 mA/cm^2^	[121]
PPy/Mxene/GA	1 M H_2_SO_4_	94%@2000	657.64	1	[126]
PANI-WO_3_/MXene	1 M H_2_SO_4_	82%@3000	741	1	[130]
rGO/MXene-PPy	1 M H_2_SO_4_	67.3%@10,000	408.2	10	[134]
MXene@h-CNT	2 M KOH	80%@4000	404	4	[139]
MXene/rGO/CNTs	1 M H_2_SO_4_	92.9%@8000	463.5	1	[145]
CF-PhNO_2_/oPD-MX	1 M H_2_SO_4_	94%@5000	157	5 mV/s	[148]
MoSSe@CNTs	1 M H_2_SO_4_	-	585	-	[153]
NF@Mxene@NiCo-LDH	3 M KOH	87%@10,000	22.6 F/cm^2^	5 mA/cm^2^	[159]
Ni-MOF/MXene	1 M KOH	87.20%@2000	716.19	1	[166]
FeCo-LDH/MXene	3 M KOH	82.4%@5000	2058.2	1	[170]
MXene/BCN_10_ (m-MX/BCN_10_)	KOH	95%@10,000	678	0.5	[173]
MXene/Ni-Co phosphide	2 M KOH	93.8%@10,000	1754	3 mA/cm^2^	[179]

**Table 2:** Sensing performance of the MXene-based electrode materials for the determination of different analytes.

Electrode Modifier	Sensing Method	LOD	Linear Range	Sensitivity	Analyte	Refs.
ZnO TPs/MXene	CA	17 μM	0.05 to 0.7 mM	29 μA mM^−1^ cm^−2^	Glu	[180]
MXene/V_2_O_5_	DPV	87 nM	414 nM to 31.2 µM	-	BPA	[181]
Nb_2_C/MnFe_2_O_4_	DPV	0.079 μM	0.1 to 1000 μM	-	AP	[182]
Nb_2_C/MnFe_2_O_4_	DPV	0.070 μM	0.1 to 60 μM	-	DA	[182]
Cu_2_O/MXene/rGO	CA	1.1 μM	0.1 to 14 and 15 to 40 mM	264.52 μA mM^−1^ cm^−2^	Glu	[183]
Cu_2_O/MXene/AC	CA	1.96 μM	0.004 to 13.3 mM and 15.3 to 28.4 mM	430.3 μA mM^−1^ cm^−2^	Glu	[184]
Au@Ce_2_Sn_2_O_7_/MXene	DPV	5.63 nM	0.00125 to 1021.96 μM	0.403 μA·μM^–1^·cm^–2^	MTL	[185]
ZnMoO_4_/MXene	DPV	0.0081 μM	10.65 to 605.65 μM	10.413 μA·μM^–1^·cm^–2^	ROX	[186]
AChE-CS/CMVON	DPV	2.3 × 10^−14^ M	3.6 × 10^−13^ to 3.6 × 10^−8^ M	-	fenitrothion	[187]
NbC@Mo NC	DPV	1.5 × 10^−10^ M	1.0 × 10^−6^ to 1.9 × 10^−3^ M	-	fenitrothion	[187]
MIP/CFO/MXene/MCPE	SWV	1.6 nM	0.005 to 0.7 μM and 0.7 to 10 μM	-	quercetin	[188]
ZnMoO_4_/MXene	DPV	12 CFU/ml	10 to 10^7^ CFU/ml	-	L. monocytogenes	[190]
CuO-CeO_2_/MXene	CA	1.67 μM	5.0 to 100 μM	-	H_2_O_2_	[191]
MXene/MoS_2_	CA	4.2 μM	-	54.6 nAμM^−1^	AA	[192]
CFP-MXene-MoS_2_	CA	1.47 μM	10 to 3000 μM	-	AA	[193]
CFP-MXene-MoS_2_	CA	0.27 μM	0.5 to 1000 μM	-	DA	[193]
CFP-MXene-MoS_2_	CA	0.38 μM	0.5 to 1000 μM	-	UA	[193]
AChE-Chit/Pt/MoS_2_/TM	DPV	4.71 × 10^−13^ M	10^−6^ to 1 μM	-	chlorpyrifos	[194]
Mxene-AgBiS_2_	DPV	2.54 nM	0.02 to 5 and 10 to 78 μM	-	4-NP	[195]
*N*-MPG/CuS flower-like/MXene	DPV	1.6 μM	5 to 150 μM	-	NAL	[196]
Ti_3_C_2_MXene/MoS_2_@AuNPs/AChE	DPV	5.29 × 10^−15^ M	1 × 10^−13^ to 1 × 10^−7^ M	-	phoxim	[197]
LOx/Pt/PANI/MXene	Amperometry	5.0 μM	0.005 to 5 mM	-	lactate	[198]
PANI-Ti_3_C_2_	ASV	0.017 μg/L	0.1 to 20 μg/L	-	Hg^2+^	[199]
MIP/pTHi/MXene/Fe@Ti-MOF-NH_2_	SWV	0.54 μM	0.1 to 4000 μM	-	CC	[200]
(P_2_Mo_17_V/Cs-Ti_3_C_2_T_x_)_2_	DPV	0.08 μM	0.1 to 103 μM	0.141 μA·μM^–1^·cm^–2^	L-Trp	[201]
Ti_3_C_2_T*_x_*	DPV	0.031 µM	10 to 500µM	564.30 μA mM^−1^ cm^−2^	Glu	[202]
Ti_3_C_2_T_x_/poly(rutin)	DPV	1 nM	1.0 × 10^−9^ to 1.0 × 10^−4^ M	0.49 μA·μM^–1^·cm^–2^	ciprofloxacin	[203]
Ti_3_C_2_-MWCNT	DPV	0.0066 µM	2 to 150	-	HQ	[204]
Ti_3_C_2_-MWCNT	DPV	0.0039 µM	2 to 150	-	CC	[204]
MXene@PDA/NH_2_-MWCNTs	DPV	1 nM	0.005 to 10.0 and 10.0 to 60.0 µM	-	AP	[206]
Ti_3_C_2_@N-C	SWASV	2.55 nM	0.1 to 4 µM	49.85 μM μA^−1^	Cd^2+^	[207]
Ti_3_C_2_@N-C	SWASV	1.10 nM	0.05 to 2 µM	177.33 μM μA^−1^	Pb^2+^	[207]
Ti_3_C_2_/G-MWCNTs/ZnO	DPV	3.2 nM	0.01 to 30 µM	16 A/M	DA	[208]
3D MGMA	DPASV	0.48 μg L^−1^	3 to 900 μg L^−1^	-	Zn^2+^	[209]
3D MGMA	DPASV	0.45 μg L^−1^	3 to 900 μg L^−1^	-	Cd^2+^	[209]
3D MGMA	DPASV	0.29 μg L^−1^	3 to 900 μg L^−1^	-	Pb^2+^	[209]
Pt@SWCNTs-Ti_3_C_2_-rGO	DPV	2.5 nM	0.006 to 11.4 µM	1.941 μA (μmol L^−3^)^−1^ cm ^−2^	BPA	[210]
N, S-CDs/Ti_3_C_2_T_x_	DPV	0.91 μM and 3.71 μM	1 to 1000 μM	-	DA	[213]
Ti_3_C_2_:GQDs(1:3)	DPV	1.8 μM	40 to 400 μM	-	DA	[215]
layered N-doped carbon/MXene	SWASV	0.019 μM	-	114.54 µA µM^−1^ cm^−2^	Cu^2+^	[216]
layered N-doped carbon/MXene	SWASV	0.056 μM	-	64.317 µA µM^−1^ cm^−2^	Hg^2+^	[216]
Ce-MOF/Ti_3_C_2_T_X_ MXene	DPV	0.19 μM	0.2 to 139 μM	-	L-Trp	[218]
MOF-Ti_3_C_2_	DPV	110 nM	90 to 130 nM	-	DA	[220]
Fe-MOF-NH_2_/CNTs-NH_2_/MXene	DPV	13.2 nM	0.1 to 100 μM	-	ofloxacin	[222]
MXene-NH_2_@CeFe-MOF-NH_2_	DPV	0.95 nM	5 to 50 nM	-	Pb^2+^	[224]
MXene-NH_2_@CeFe-MOF-NH_2_	DPV	0.32 nM	1 to 35 nM	-	Hg^2+^	[224]
MXene@PDA/MOF	DPV	0.00374 μM	0.01 to 5 μM	-	L-Cys	[226]
Ru/NiFe-LDH-MXen	LSV	2.2 nM	0.01 to 275 μM	152.44 μA·μM^–1^·cm^–2^	NFT	[227]
FeCu-LDH@MXene	DPV	0.09 μM	0.66 to 418 μM	-	CLZP	[228]
PtNP@MXene-Ti_3_C_2_T_x_	AMP	0.45 μM	10 to 110 μM	1.5906 nA/μM	L-glutamate	[229]
MXene-Ni NPs	DPV	0.12 pM	0.001 to 0.017 μM	-	MMA	[232]
MO/Ti_3_C_2_	CA	0.05 μM	0.33 to 1200 μM	-	H_2_O_2_	[234]
MO/Ti_3_C_2_	CA	0.01 μM	0.1 to 1350 μM	-	N_2_H_4_	[234]
